# Impact of Pore Size and Defects on the Selective Adsorption of Acetylene in Alkyne‐Functionalized Nickel(II)‐Pyrazolate‐Based MOFs

**DOI:** 10.1002/chem.202100821

**Published:** 2021-07-09

**Authors:** Farzaneh Afshariazar, Ali Morsali, Simona Sorbara, Natalia M. Padial, Esther Roldan‐Molina, J. Enrique Oltra, Valentina Colombo, Jorge A. R. Navarro

**Affiliations:** ^1^ Department of Chemistry Faculty of Sciences Tarbiat Modares University P.O. Box 14115-175 Tehran Iran; ^2^ Department of Chemistry Università degli Studi di Milano Via Golgi, 19 20133 Milano Italy; ^3^ Departamento de Química Universidad de Granada Av. Fuentenueva S/N 18071 Granada Spain; ^4^ Unidad de Excelencia de Química Universidad de Granada Av. Fuentenueva S/N 18071 Granada Spain

**Keywords:** breakthrough curves, carbon capture, gas separation, computational modelling, temperature swing adsorption

## Abstract

C_2_H_2_/CO_2_ separation is a highly challenging process as a consequence of their similar physicochemical properties. In this work we have explored, by static and dynamic gas sorption techniques and computational modelling, the suitability of a series of two isoreticular robust Ni(II)pyrazolate‐based MOFs, bearing alkyne moieties on the ligand backbones, for C_2_H_2_/CO_2_ separation. The results are consistent with high adsorption capacity and selectivity of the essayed systems towards C_2_H_2_ molecules. Furthermore, a post‐synthetic treatment with KOH ethanolic solution gives rise to linker vacancy defects and incorporation of extraframework potassium ions. Creation of defects is responsible for increased adsorption capacity for both gases, however, strong interactions of the cluster basic sites and extraframework potassium cations with CO_2_ molecules are responsible for a lowering of C_2_H_2_ over CO_2_ selectivity.

## Graphical abstract

**Robust defective** nickel(II) pyrazolate‐based MOFs, bearing alkyne moieties on the organic spacers have been explored for C_2_H_2_/CO_2_ separation. As proven by static and dynamic gas adsorption and computational modelling the non‐defective34 systems give rise to selective interactions with C_2_H_2_ over CO_2_ as a result of a mixture of pore confinement and H‐bonding and alkyne‐alkyne interactions. Creation of defects is responsible for strong interactions of cluster basic sites and extraframework potassium cations with CO_2_ with a concomitant lowering of C_2_H_2_ over CO_2_ selectivity.

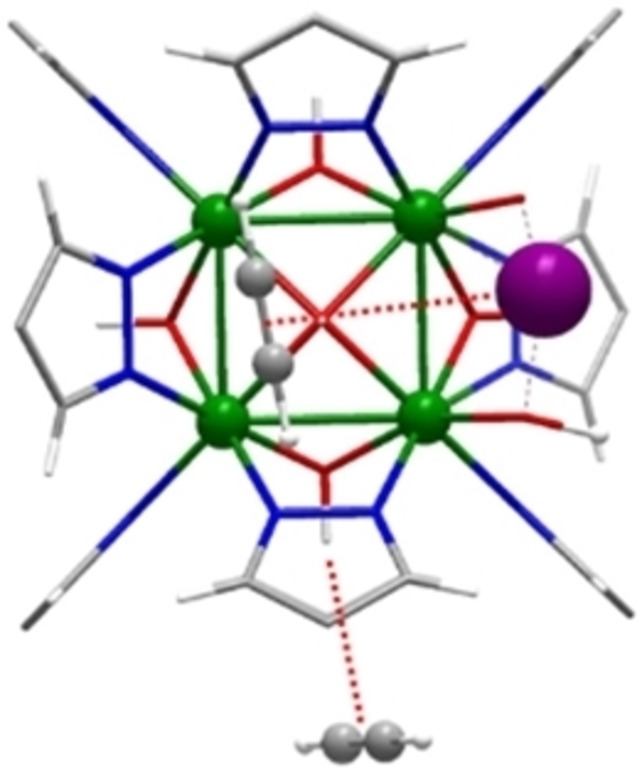

## Introduction

Gas separation is one of the major challenging industrial processes due to high energy consumption of traditional thermal separation procedures.^[1]^ Despite of the considerable progress in separation technologies, the development of high‐performance alternatives for gas separation processes with lower energy penalty, such as adsorption, is regarded as a serious priority that can help to mitigate global warming.^[2]^

In recent years, Metal‐organic frameworks (MOFs) and related materials have emerged as a fascinating group of advanced functional porous materials due to their unprecedented modular nature in both chemical composition and pore properties engineering.^[3]^ Systematic variation of their primary building blocks, rational design of proper functional sites, accessible channels, and uniform distribution of their recognition sites as well as post‐treatment approaches offer a plethora of opportunities to overcome the limitations of traditional adsorptive materials.[^4^, ^5^] Among the various gas separation processes, the separation of C_2_H_2_/CO_2_ gas mixtures is one of the most challenging processes. The difficulty of this separation is mainly due to the similar physicochemical characteristics of these two gas molecules.^[6]^ Indeed, the unavoidable amount of CO_2_ impurities, generated during the production of C_2_H_2_, need to be efficiently removed from the product gas, to obtain high quality C_2_H_2_. Pure C_2_H_2_ is then mostly used in industry as a valuable fuel gas, as well as, a major feedstock for the production of various industrial petrochemicals and electrical materials.^[7]^ The current technologies for acetylene purification are nowadays the extraction with organic solvents or the cryogenic distillation processes, which result both in adverse environmental impacts and high energy consumption.^[8]^ In this regard, the selective physisorption process within functionalized MOFs can be regarded as a promising solution to the all above mentioned problems. To date, a few numbers of MOFs have been purposefully designed as suitable platforms for acetylene purification and storage.[^9^, ^10^, ^11^, ^12^] Various strategies have been applied to provide effective host‐guest interactions within the framework. Among them, functionalization of pore surfaces along with post‐modification treatments can result in a high gas adsorption capacity and selectivity to a gas molecule.^[13]^ A few systematic investigations have revealed that alkyne‐functionalized MOFs demonstrate higher adsorption capacity along with desired selectivity for acetylene gas molecules in comparison to their non‐functionalized analogues.[^14^, ^15^] Along with these observations, as demonstrated by previous work of some of us, the exploitation of pyrazolate‐based spacers can afford remarkable chemical and thermal stability to MOFs, often exceeding 400 °C in air.[^16^, ^17^] Moreover, the introduction of structural defects in MOFs has been shown to be a very powerful strategy to enhance and modulate the adsorption behavior and gas separation performances of the material.[^18^, ^19^, ^20^] A representative example is the impact of defects on CO_2_/H_2_O adsorptive separation on defective UiO‐66.^[21]^ However, to the best of our knowledge the impact of presence of defects on acetylene adsorption and separation has not been explored.

**Scheme 1:**
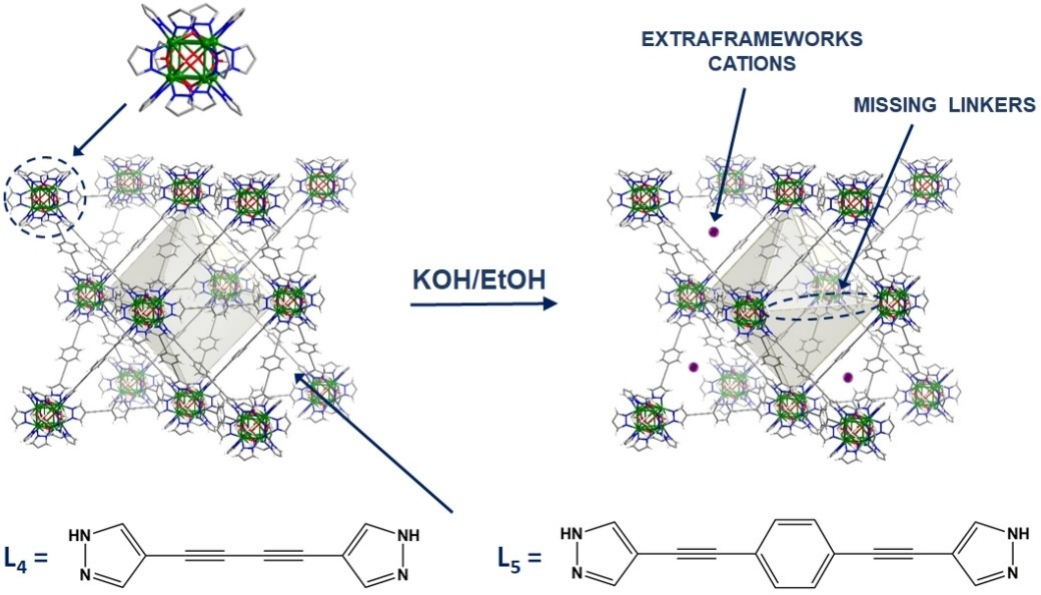
Schematic representation of post‐synthetic treatment of [Ni_8_(OH)_4_(H_2_O)_2_(L_5_)_6_] ([Ni_8_(L)_6_]) systems with KOH to yield K[Ni_8_(OH)_6_(L_5_)_5.5_] ([Ni_8_(L)_6_]@K) defective materials.

Taking into account this background we have selected two isoreticular robust Ni‐bispyrazolate MOFs, namely, [Ni_8_(OH)_4_(H_2_O)_2_(L_4_)_6_] and [Ni_8_(OH)_4_(H_2_O)_2_(L_5_)_6_], where L_4_=(4,4′‐buta‐1,3‐diyne‐1,4‐diyl)bis‐pyrazole, and L_5=_4,4′‐(benzene‐1,4‐diyldiethyne‐2,1‐diyl)bis‐pyrazole, previously reported by us,^[17]^ bearing alkyne functional groups on the linkers’ backbones as possible preferential adsorption sites for acetylene molecule (Scheme 1). Moreover, post‐synthetic treatment modification has been applied to the samples, to create additional potential interaction sites within the frameworks via partial removal of the bridging linkers to yield defective K[Ni_8_(OH)_6_(L)_5.5_] ([Ni_8_(L)_6_]@K) systems (Scheme 1). This postsynthetic modification with KOH is expected to facilitate the diffusion of the gas molecules into the framework and to increase the number of preferential adsorption sites. Further, we have studied the impact of these structural features in the challenging separation of C_2_H_2_/CO_2_ gas mixtures by a combination of static gas sorption techniques, dynamic pulse gas chromatography, breakthrough experiments and computational modelling.

## Results and Discussion

**Figure 1:**
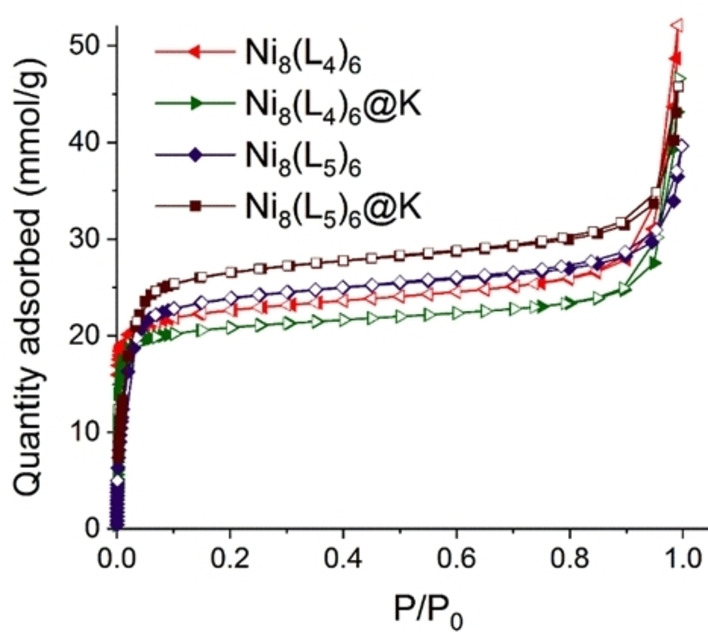
N_2_ sorption isotherm at 77 K for non‐defective [Ni_8_(L_4_)_6_], [Ni_8_(L_5_)_6_], and defective, [Ni_8_(L_4_)_6_]@K and [Ni_8_(L_5_)_6_]@K systems.

[Ni_8_(OH)_4_(H_2_O)_2_(L)_6_] MOFs (abbreviated as [Ni_8_(L)_6_], where L is a general bis‐pyrazolate linker, are based on octanuclear Ni_8_(OH)_4_(H_2_O)_2_(pyrazolate)_12_ SBUs acting as 12 connected nodes, leading to 3D *fcu* networks with highly accessible *T*_d_ and *O*_h_ voids.^[17]^ The phase purity of the bulk products were examined by Powder X‐ray Diffraction (PXRD) analyses revealing a good agreement with the simulated diffraction patterns (Figures S1–S4). Brunauer‐Emmett‐Teller (BET) surface measurements based on nitrogen sorption isotherms were obtained from N_2_ isotherms at 77 K and result to be 1990 m^2^/g and 2540 m^2^/g for [Ni_8_(L_4_)_6_] and [Ni_8_(L_5_)_6_], respectively. The unique features of these two alkyne‐functionalized Ni‐MOFs, encouraged us to explore the separation performance of the challenging C_2_H_2_/CO_2_ gas mixtures. Moreover, the effect of post‐synthetic treatment with 0.35 M KOH ethanolic solution was also investigated. As previously reported by some of us, postsynthetic modification treatment of [Ni_8_(OH)_4_(H_2_O)_2_(1,4‐bipyrazolatebenzene)_6_] with KOH ethanolic solution leads to the creation of linker vacancy defects within the MOF structures with a concomitant higher pore accessibility and adsorption interactions.^[19]^ In this work, we have studied if this strategy could be extended to the alkyne functionalized isoreticular systems [Ni_8_(OH)_4_(H_2_O)_2_(L_4_)_6_] and [Ni_8_(OH)_4_(H_2_O)_2_(L_5_)_6_], where L_4_=(4,4′‐buta‐1,3‐diyne‐1,4‐diyl)bis‐pyrazole, and L_5=_4,4′‐(benzene‐1,4‐diyldiethyne‐2,1‐diyl)bis‐pyrazole which after KOH treatment yield defective K[Ni_8_(OH)_6_(L)_5.5_] ([Ni_8_(L)_6_]@K) systems. To evaluate the effect of this post‐synthetic treatment on the materials’ properties, we firstly measured N_2_ adsorption isotherms over the pristine materials. The results are indicative of a diminution of the specific BET surface upon passing from pristine [Ni_8_(L)_6_] materials to defective [Ni_8_(L)_6_]@K systems (Figure 1 and Table 1). By contrast, DFT analysis of the pore size distribution is indicative of increased accessibility to the larger pores. Thus, while for [Ni_8_(L_4_)_6_] the porosity is mainly due to 1.2 nm pore voids with a small share of the 1.5 nm voids for [Ni_8_(L_4_)_6_]@K the porosity is equally distributed among 1.1 and 1.5 nm pores (Figure S5a,b). Similarly, for [Ni_8_(L_5_)_6_], it is also possible to appreciate an increased accessibility to the 2.3 nm mesopores in [Ni_8_(L_5_)_6_]@K (Figure S5c, d). In addition, PXRD patterns and structureless Le Bail refinements revealed that the integrity of the structures has retained after the post‐treatment modification due to the significant robustness of the Ni‐bispyrazolate frameworks (Figures S1–S4).

**Table 1:** Adsorption data of Ni‐MOFs for C_2_H_2_ and CO_2_ gas molecules at 298 K.

α_C2H2/ CO2_^[f]^	C_2_H_2_/CO_2_ Uptake^[e]^	IAST Selectivity^[c/d]^	C_2_H_2_/CO_2_ Uptake^[b]^	Q_st_ C_2_H_2_/CO_2_^[a]^	S_BET_	Ni‐MOF
4.18	1.59/0.38	3.4/2.3	1.7/0.4	29.5/23.0	1990	[Ni_8_(L_4_)_6_]
3.13	2/0.64	3.3/2.57	2.6/0.88	29.0/27.2	1850	[Ni_8_(L_4_)_6_]@K
3.87	1.2/0.31	2.55/1.78	1/0.3	25.6/11.9	2540	[Ni_8_(L_5_)_6_]
3.35	2.18/0.65	2.57/1.94	2.0/0.52	27.1/28.0	2165	[Ni_8_(L_5_)_6_]@K

[a] Calculated from the Clausius‐Clapeyron equation at 273 and 298 K (KJ/mol); [b] Uptake capacity obtained at 0.15 bar and 298 K from single‐component isotherm (mmol/g); [c] Selectivity determined from IAST for an equimolar mixture of C_2_H_2_/CO_2_ at 0.15 bar and 298 K.; [d] Selectivity determined from IAST for an equimolar mixture of C_2_H_2_/CO_2_ at 1 bar and 298 K.; [e] Uptake capacity obtained at 298 K from breakthrough measurements (mmol/g); [f] Selectivity determined from breakthrough measurements at 298 K

### Gas adsorption studies

To investigate the gas separation performance of the as‐synthesized Ni‐MOFs as well as to evaluate the impact of the introduction of linker vacancy defects within the structures, static gas adsorption isotherms, dynamic breakthrough measurements, along with the pulse gas chromatographic studies were performed.

### Static gas sorption studies

**Figure 2:**
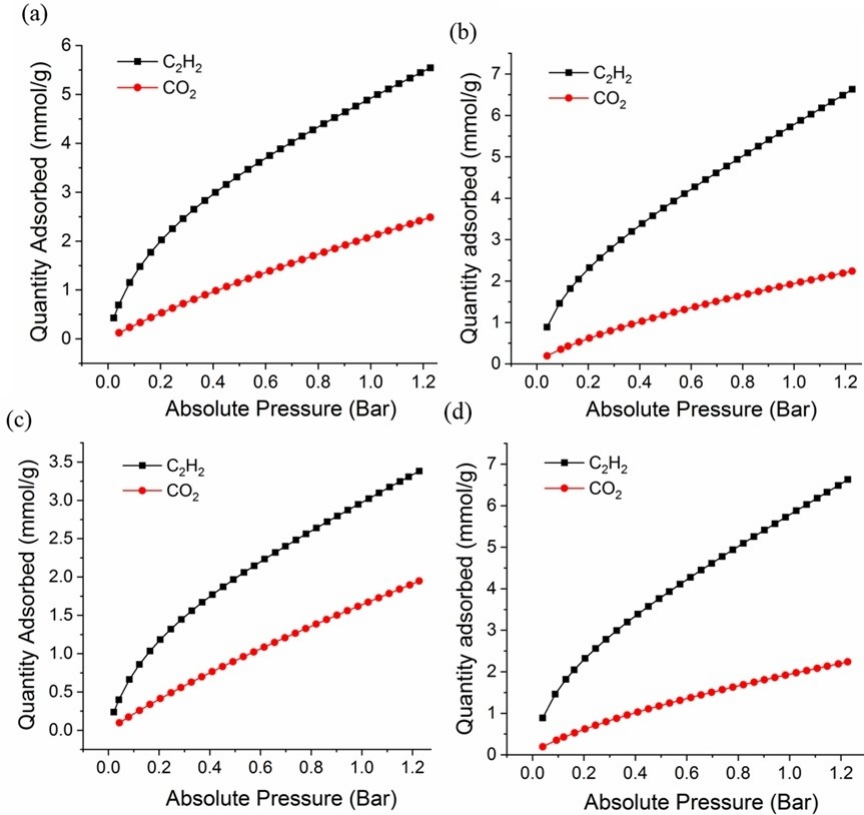
Gas sorption isotherms of [Ni_8_(L_4_)_6_] (a); [Ni_8_(L_4_)_6_]@K (b); [Ni_8_(L_5_)_6_] (c) and[Ni_8_(L_5_)_6_]@K (d) for C_2_H_2_ and CO_2_ at 298 K.

Static gas sorption data for C_2_H_2_ and CO_2_ gas molecules were collected at 273 and 298 K (Figures 2 and S5–S8). As illustrated in Figure 2 the uptake capacity of C_2_H_2_ molecules in all Ni‐MOF structures is approximately twice of the adsorption amount of CO_2_ molecules being indicative of high selectivity towards C_2_H_2_ gas molecules (see below). This result can be attributed to the presence of the alkyne functional group within the framework, resulting in much more effective interaction with C_2_H_2_.[^14^, ^15^] Proper pore size match is another key factor that provides effective interactions with guest molecules within the structures. [Ni_8_(L_4_)_6_] with microporous nature (1.26 nm pore size) demonstrated much higher C_2_H_2_ uptake amount than [Ni_8_(L_5_)_6_] (1.85 nm and 2.2 nm for Td and Oh voids, respectively) due to proper pore size match of the former material, leading to much more effective interactions with C_2_H_2_ molecules. It is worth to mention that the C_2_H_2_ uptake value of [Ni_8_(L_4_)_6_] (5.0 mmol/g at 298 K) is among the highest on well‐known MOFs with specific interaction sites (see Table 2).

**Table 2:** Adsorption data of representative MOFs for C_2_H_2_ uptake at 1 bar.

Material	SBET [m^2^g^−1^]	C_2_H_2_ uptake [mmolg^−1^]	T_ad_ [K]	Ref.
[Ni_8_(L_4_)_6_]	1990	5.0	298	This work
[Ni_8_(L_4_)_6_]@K	1850	6.0	298	This work
[Ni_8_(L_5_)_6_]	2540	3.0	298	This work
[Ni_8_(L_5_)_6_]@K	2165	5.7	298	This work
MFM‐137	1749	9.28	273	[15]
UPC‐200(Fe)‐F‐BIM	2213	6.24	298	[12]
ATC‐Cu	600	5.01	298	[26]
MIL‐100(Fe)	2300	5.3	293	[27]
UTSA‐74	830	4.8	298	[28]
UTSA‐300	311	3.41	273	[29]
FJU‐22	828	5.1	296	[30]
TIFSIX‐2‐Cu‐i	685	4.1	298	[31]
JCM‐1	550	3.3	298	[32]
MUF‐17	247	3.01	293	[33]
ZJUT‐2	350	3.30	293	[34]
NKMOF‐1‐Ni	382	2,72	298	[35]
Cu_2_(pzdc)_2_(pyz)	571	1.88	300	[36]

Moreover, the results showed that the introduction of linker vacancy defects gives rise to higher adsorption capacities. Indeed, KOH treatment of [Ni_8_(L_5_)_6_] to yield [Ni_8_(L_5_)_6_]@K gives rise to increments on adsorption capacity of 90 % and 63 % for C_2_H_2_ and CO_2_ molecules at 298 K, respectively (Figure 2c,d). The adsorption capacity for C_2_H_2_ at 298 K and 1 bar found for [Ni_8_(L_4_)_6_]@K and [Ni_8_(L_5_)_6_]@K of 6.0 and 5.7 mmol/g, respectively, are among the highest reported so far (see Table 2). The enhancement of adsorption capacity can be regarded as a result of the increased number of adsorption sites arising from missing linker defects and extraframework K^+^ cations. Indeed, charge gradients created by extraframework K^+^ cations might be responsible for providing suitable interaction sites with adsorbates possessing quadruple moments (Scheme 1).^[22]^ In addition, deprotonation of the coordinated water molecules of [Ni_8_(OH)_4_(H_2_O)_2_] to [Ni_8_(OH)_6_] metal clusters leads to increased basicity.^[19]^

For further investigation, the isosteric heat of adsorption (Q_st_) was calculated to evaluate the interaction strength between the adsorbates and the Ni‐MOF pore surface. The calculation was performed based on the Clausius‐Clapeyron equation (Eq. (1)) derived from the gas sorption isotherms obtained at 273 and 298 K.

(1) $$Q{}_{\mathrm{st}}=-R\left[\Delta \left(lnP\right)/\Delta \left(1/T\right)\right]{}_{N}$$

The calculated Q_st_ values are given in Table 1. The higher Q_st_ values for C_2_H_2_ over CO_2_ on [Ni_8_(L_4_)_6_] and [Ni_8_(L_5_)_6_] systems can explain the observed selectivity towards C_2_H_2_. As illustrated in Figures S10 and S11, by increasing the pressure value, the Q_st_ value decreases, which can be attributed to the weaker interaction between the adsorbates and the adsorbents as a consequence of the covering of the most active adsorption sites on the pore surface.[^23^, ^24^] Noteworthy, the Q_st_ values for the C_2_H_2_ are slightly modified upon the creation of defects with a small decrease for [Ni_8_(L_4_)_6_]@K and a small increase for [Ni_8_(L_5_)_6_]@K. By contrast, the Q_st_ values for the CO_2_ increase significantly upon the creation of defects (Table 1 and Figures S10, S11). This result can be attributed to CO_2_ stronger interactions with extraframework K^+^ cations and framework basic sites (see below) and can justify the observed decrease of selectivity of C_2_H_2_ over CO_2_ (Table 1). We here suggest that, the creation of a high density of basic OH sites and extraframework K^+^ ions, after post‐treatment modification, provides more effective interaction sites for CO_2_ binding (see below), leading to higher Q_st_ values for this gas molecule in both defective systems.^[25]^

Further investigation was performed by calculation of the adsorption selectivity from the pure single‐component isotherms obtained at 298 K based on IAST Theory.^[37]^ The experimental results were well fitted to the dual‐site Langmuir‐Freundlich equations (Figures S12–S19). The fitting parameters were used to calculate the adsorption selectivity based on IAST. The C_2_H_2_/CO_2_ selectivity values for an equimolar gas mixture (50/50 v/v) at 0.15 and 1 bar are given in Table 1 and Figure S20. The results obtained at 298 K and 0.15 bar agree with the observed highest C_2_H_2_ selectivity in microporous [Ni_8_(L_4_)_6_] as a result of a suitable pore size match and presence of alkyne residues (see below). Moreover, IAST theory calculations were applied to evaluate the behavior of the separation process at higher pressures. The results are indicative of a 25–32 % drop in selectivity values of C_2_H_2_ over CO_2_ upon increasing the components pressure from 0.15 to 1 bar. This behavior can be related to a probable covering of the most active adsorption sites at lower pressures with a concomitant loss of selective interaction sites.

### Dynamic gas sorption studies

Advanced gas sorption studies including dynamic breakthrough techniques, in addition to pulse gas chromatographic experiments, were also performed to show the behavior of the pristine and defective materials towards complex gas mixtures.

### Breakthrough curves

**Figure 3:**
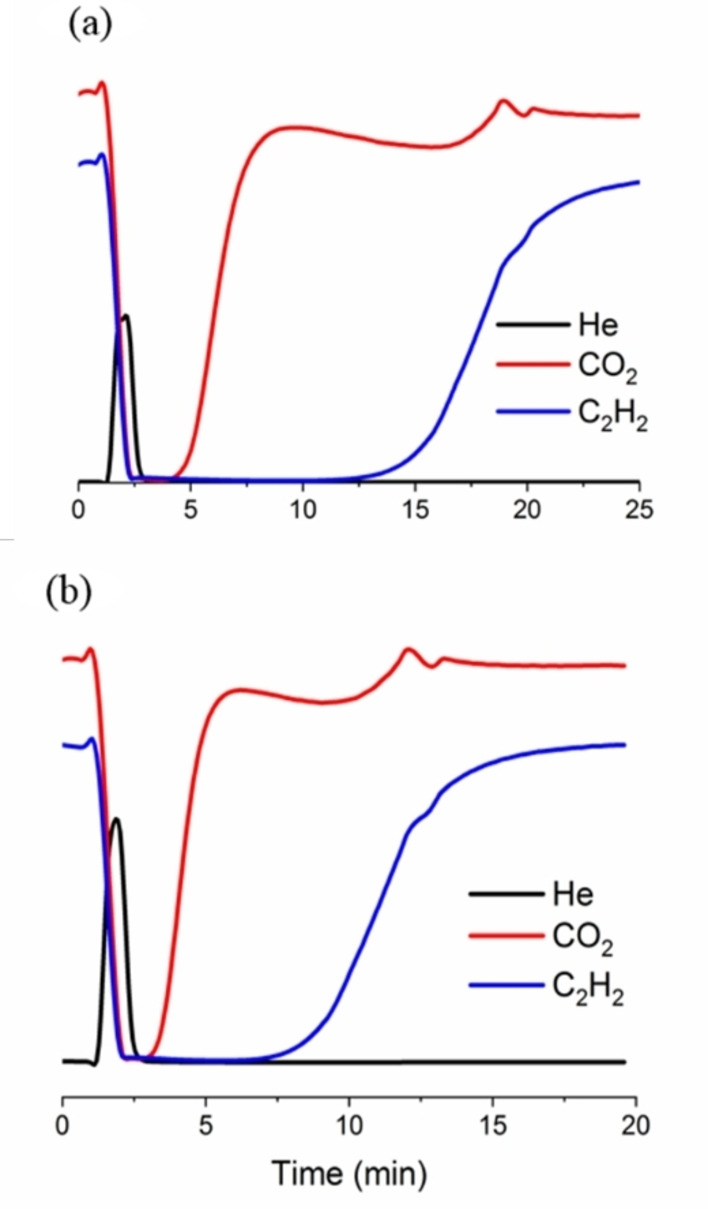
Breakthrough curves of [Ni_8_(L_4_)_6_]@K: a) 273 K, b) 298 K for a gas mixture of C_2_H_2_/CO_2_/N_2_ (3 : 3 : 14 mL min^−1^). The observed perturbation in the shape of the breakthrough curves (17 min (a), 12.5 min (b)) can be regarded to a roll‐up phenomenon of the weaker adsorbate (CO_2_) being desorbed by the strongly interacting adsorbate (C_2_H_2_).

To evaluate the practical performance of Ni‐MOFs for C_2_H_2_/CO_2_ gas separation, measurements of dynamic breakthrough curves were done at 273 and 298 K. A gas mixture of C_2_H_2_, CO_2_, and N_2_ with the volumetric composition of 3 : 3 : 14 mL min^−1^, respectively was flowed through a fixed‐bed column packed with the desired adsorbent. The breakthrough curves are given in Figures 3, and S21–S23. As expected [Ni_8_(L_4_)_6_] MOF revealed higher adsorption capacity towards C_2_H_2_ molecules in comparison to [Ni_8_(L_5_)_6_] MOF which can be related to the micropore size of the former structure leading to more effective adsorbate‐adsorbent interactions.^[38]^ Noteworthy, C_2_H_2_ over CO_2_ selectivity values of 4.18 and 3.87, respectively for [Ni_8_(L_4_)_6_] and [Ni_8_(L_5_)_6_] systems are higher than the ones calculated from IAST theory (see above) which points to the difficulty of predicting the behavior of a complex gas mixture from single component adsorption isotherms.

The C_2_H_2_/CO_2_ separation performance of [Ni_8_(L_4_)_6_]@K and [Ni_8_(L_5_)_6_]@K MOFs were also investigated to evaluate the impact of introduction of missing linker defects within the frameworks. The results showed that the gas adsorption capacities increase significantly. An increment of about 26 % of adsorption capacity towards C_2_H_2_ gas molecules was observed on passing from [Ni_8_(L_4_)_6_] to [Ni_8_(L_4_)_6_]@K. In the case of [Ni_8_(L_5_)_6_] the KOH treatment give rise to 82 % increase for [Ni_8_(L_5_)_6_]@K (Figures S22, S23). Noteworthy, post‐synthetic modification with KOH ethanolic solution has a higher impact on CO_2_ adsorption capacity in both [Ni_8_(L_4_)_6_] and [Ni_8_(L_5_)_6_] systems with 68 % and 110 % increments for [Ni_8_(L_4_)_6_]@K and [Ni_8_(L_5_)_6_]@K systems, respectively. The higher enhancement on the CO_2_ adsorption capacity in comparison with C_2_H_2_ gives rise to a slight decrease of the selectivity towards C_2_H_2_ over CO_2_ to 3.13 and 3.35 for [Ni_8_(L_4_)_6_]@K and [Ni_8_(L_5_)_6_]@K, respectively, agreeing with the observed lower impact of defects on Q_st_ values for C_2_H_2_ in comparison to CO_2_. Noteworthy, the obtained uptake capacities for C_2_H_2_ and CO_2_ molecules from breakthrough measurements are comparable to those obtained from single‐component isotherms confirming the consistency of the results.

### Variable‐temperature pulse chromatographic studies

To further examine the interaction strength of the adsorbates with the framework, a gas mixture of equimolecular quantities of C_2_H_2_, CO_2_, and H_2_ gas molecules was injected to gas chromatograph equipped with a fixed‐bed column packed with the desired adsorbent. The experiments were performed at 273–248 K, and He was used as an inert carrier gas with a flow of 20 mL min^−1^. *Q_st_* was calculated by Clausius‐Clapeyron equation (Eq. (1)).

Henry constant values were also calculated by using V_g_ (retention volume) as a function of the temperature. The order of the retention volume was measured to be as follow: C_2_H_2_>CO_2_>H_2_ (Figures S23–S26). A negligible interaction was observed for H_2_ molecules with the structures which could be considered as a reference for the dead volume of the column. The results demonstrated that higher interactions are at work in the defective MOFs [Ni_8_(L_4_)_6_]@K and [Ni_8_(L_5_)_6_]@K, which should be related to interaction with the most active sites at low coverage (Table S1).

### Molecular simulations

**Figure 4:**
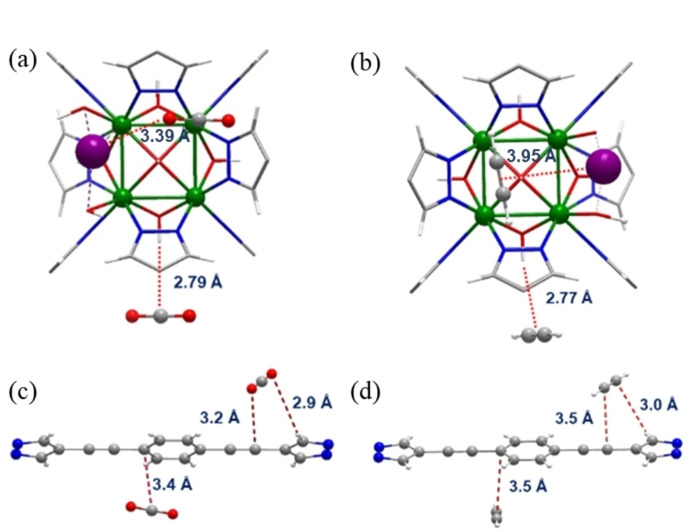
Depiction of major interactions observed from the adsorption locator module of Materials Studio (7.0) for CO_2_ (a and c) and for C_2_H_2_ (b and d).

Aiming at unveiling the CO_2_ and C_2_H_2_ adsorption mechanism, two theoretical models, considering 6 and 54 molecules of adsorbate molecules per unit cell, for the non‐defective [Ni_8_(L_4_)_6_] and [Ni_8_(L_5_)_6_] systems were obtained using the adsorption locator module of Materials Studio (7.0) on a crystal cell with *P*1 symmetry.^[39]^ Firstly, the simulation with 6 guest molecules per unit cell, was used to model the primary binding sites (low pressure) of guest molecules with the host [Ni_8_(L_4_)_6_] and [Ni_8_(L_5_)_6_] frameworks. The results are indicative of short side‐on H‐bonding contacts between the metal cluster OH groups and the guest molecules [OH⋅⋅⋅C_CO2_=2.79 Å; OH⋅⋅⋅C≡C_C2H2_=2.77 Å] (Figure 4a, 4b). The simulations at higher guest loadings are indicative of a wider variety of interactions taking place between the guest molecules and the host framework. The most common interactions for CO_2_ are between the benzene [C_benzene_⋅⋅⋅C_CO2_=3.4 Å], alkyne [C_ethine_⋅⋅⋅O_CO2_=3.2 Å] and pyrazole residues [H_pyrazole_⋅⋅⋅O_CO2_=2.9 Å] (Figure 4c). Similarly, for C_2_H_2_ the most common interactions are between the benzene ring [C_benzene_⋅⋅⋅C_C2H2_=3.5 Å], alkyne [C_alkyne_⋅⋅⋅C_C2H2_=3.5 Å] and pyrazole residues [H_pyrazole_⋅⋅⋅C_C2H2_=3.0 Å] (Figure 4d). The results are indicative of the cooperative impact of basic hydroxide residues, and unsaturated organic residues in giving rise to strong interactions of the guest molecules with the host framework. Additionally, we have also modelled the interaction of the adsorbate molecules with defective [Ni_8_(L_4_)_6_]@K and [Ni_8_(L_5_)_6_]@K systems, using a geometry optimized model in which one of the bipyrazolate linkers in the crystal cell has been replaced by four hydroxide groups coordinated to two adjacent metal clusters and two extraframework potassium ions. A computational modelling of the adsorption process reveals that the removal of the linkers lead to preferential accommodation of C_2_H_2_ molecules in non‐defective cavities as a consequence of higher confinement effects. On the other hand, it is also possible to observe significant shorter contacts of the extraframework potassium ions with the CO_2_ adsorbate molecules [K⋅⋅⋅O_CO2_=3.39 Å] in comparison with the C_2_H_2_ adsorbate molecules [K⋅⋅⋅C_C2H2_=3.95 Å] (Figure 4a, b). The latter observation can be explained on the basis of the softer Lewis base nature of C_2_H_2_ in comparison to CO_2_. These results justify the observed lower impact of creation of defects on Q_st_ values for C_2_H_2_ and the concomitant lowering of selectivity towards C_2_H_2_ over CO_2_ upon defect creation (see above).

## Conclusion

In this work two isoreticular robust nickel(II) pyrazolate MOFs functionalized with alkyne functional groups were selected and their gas adsorption performance evaluated. [Ni_8_(L_4_)_6_] and [[Ni_8_(L_5_)_6_] revealed high adsorption capacity as well as high selectivity for C_2_H_2_ over CO_2_ gas molecules which can be attributed to the effective interactions with metal cluster hydroxide, aromatic and alkyne residues within the structure. Furthermore, post‐modification treatment with ethanolic solutions of KOH was performed leading to linker vacancy defects and extraframework K^+^ ions within the structures. Evaluation of the impact of linker vacancy defects in gas sorption, revealed that the adsorption capacity increases significantly as a results of higher pore accessibility, while adsorption selectivity towards C_2_H_2_ slightly decreases, as a result of stronger interactions of the CO_2_ molecules with extraframework potassium ions in comparison to C_2_H_2_ molecules. All these results exemplify the high impact of targeted functionalization of MOFs for specific adsorption properties, as well as highlight the importance of post‐synthetic modification leading to the creation of defects to further fine tune the adsorptive properties of this class of robust MOF materials.

## Experimental Section

### Characterization methods

All the chemicals were commercially available and used without further purification. Gas sorption studies were performed on a Micromeritics Triflex instrument. N_2_ sorption measurements were done at 77 K and 1 bar, while CO_2_ and C_2_H_2_ gas sorption measurements were carried at 273 and 298 K. Sample activation was achieved through thermal activation at 393 K for 12 h under vacuum. PXRD patterns were collected on a PANalytical Empyrean diffractometer (Bragg‐Brentano geometry) using copper radiation (Cu Kα=1.5418 Å) with an PIXcel detector, operating at 40 mA and 45 kV.

### MOF synthesis

The [Ni_8_(OH)_4_(H_2_O)_2_(L)_6_] MOFs (abbreviated as [Ni_8_(L)_6_] were prepared according to the reported procedure.^[17]^ Post‐treatment modification was carried out by suspending 0.055 mmol of each Ni‐MOF in 5.5 mL of 0.35 M ethanolic solution of KOH under an inert gas atmosphere for 16 h to yield missing linker defective K[Ni_8_(OH)_6_(L)_5.5_] (abbreviated as [Ni_8_(L)_6_]@K) systems. The samples were filtered off and washed thoroughly with absolute ethanol.

### Dynamic gas sorption techniques

Breakthrough measurements: A 7 cm stainless steel column with 7 mm of inner diameter was packed with about 1 g of the adsorbate followed by activation at 423 K for 1 h under 40 mL min^−1^ flow of He as an inert carrier gas. By applying a mass flow controller desired amount of gas mixtures (C_2_H_2_/CO_2_/N_2_: 3 : 3 : 14) with a total flow of 20 mL min^−1^ was prepared. The measurements were performed at 273 and 298 K. The column output was equipped with a Pfeiffer OmniStar mass spectrometer gas analysis to detect the corresponding ion peaks at 4 (He), 28 (N_2_), 26 (C_2_H_2_), and 44 (CO_2_) m/z value. The desorption process was performed several times at 393 K without observing a remarkable decrease in adsorption performance.

Pulse gas chromatography experiments: The same prepared column described as detailed above, was used for this investigation after activation under He flow at 423 K for 1 h. 600 μL of an equimolar mixture of H_2_, CO_2_, and C_2_H_2_ was injected through the column with a flow of He of 20 mL min^−1^. The experiments were carried out at various temperatures (273–248 K) and the column output was investigated using the mass spectrometer gas analysis.

### Computational calculations

A theoretical study on the interaction of CO_2_ and C_2_H_2_ molecules with non‐defective NiL_4_ and NiL_5_ systems was performed using Materials Studio 7.0 (MS7.0) software from Dassault Systemes. The Adsorption Locator module in MS7.0 software was employed to construct the simulated systems diminishing the crystal symmetry to *P*1 and to perform the MC simulation. The used forcefield was Universal, the charge was forcefield assigned and the summation methods were group and atom based. Monte Carlo simulation yielded several conformations of CO_2_ and C_2_H_2_ molecules in the non‐defective NiL_4_ and NiL_5_ systems. The results of the most stable conformation are presented in this work. A similar study was carried out on the defective [Ni_8_(L_4_)_6_]@K and [Ni_8_(L_5_)_6_]@K systems. With this aim a defective MOF material was constructed in which one of the bipyrazolate linkers in the crystal cell has been replaced by four hydroxide groups coordinated to two adjacent metal clusters and two extraframework potassium ions. After geometry optimization, the interactions of the defective porous framework with C_2_H_2_ and CO_2_ adsorbate molecules were studied as described above.

**Supporting Information**: spectroscopic characterization of the compounds, XRPD patterns, static and dynamic gas adsorption measurements, IAST fitting, and cif files of computational models.

## Conflict of interest

The authors declare no conflict of interest.

## Supporting information

As a service to our authors and readers, this journal provides supporting information supplied by the authors. Such materials are peer reviewed and may be re‐organized for online delivery, but are not copy‐edited or typeset. Technical support issues arising from supporting information (other than missing files) should be addressed to the authors.

Supporting Information

## References

[1] S. Sholl, R. P. Lively, Nature 2016, 532, 435. 27121824 10.1038/532435a

[2] J. Koros, C. Zhang, Nat. Mater. 2017, 16, 289–297. 28114297 10.1038/nmat4805

[3a] H. C. Zhou, J. R. Long, O. M. Yaghi, Chem. Rev. 2012, 112, 673–674; 22280456 10.1021/cr300014x

[3b] H. Furukawa, K. E. Cordova, M. O'Keeffe, O. M. Yaghi, Science 2013, 341, 1230444; 23990564 10.1126/science.1230444

[3c] Q. Wang, D. Astruc, Chem. Rev. 2020, 120, 1438–1511; 31246430 10.1021/acs.chemrev.9b00223

[3d] F. Afshariazar, A. Morsali, J. Wang, P. C. Junk, Chem. Eur. J. 2020, 26, 1355–1362; 31756257 10.1002/chem.201904436

[3e] F. Afshariazar, A. Morsali, Cryst. Growth Des. 2019, 19, 4239–4245.

[4a] J. R. Li, J. Sculley, H. C. Zhou, Chem. Rev. 2012, 112, 869–932; 21978134 10.1021/cr200190s

[4b] C. A. Trickett, A. Helal, B. A. Al-Maythalony, Z. H. Yamani, K. E. Cordova, O. M. Yaghi, Nat. Rev. Mater. 2017, 2, 17045;

[4c] X. Zhao, Y. Wang, D.-S. Li, X. Bu, P. Feng, Adv. Mater. 2018, 30, 1705189. 10.1002/adma.201705189 29582482

[5] W. Bury, A. M. Walczak, M. K. Leszczyński, J. A. R. Navarro, J. Am. Chem. Soc. 2018, 140, 15031–15037. 30351019 10.1021/jacs.8b09522

[6] K. Adil, Y. Belmabkhout, R. S. Pillai, A. Cadiau, P. M. Bhatt, A. H. Assen, G. Maurin, M. Eddaoudi Chem. Soc. Rev. 2017, 46, 3402–3430. 28555216 10.1039/c7cs00153c

[7] P. Pässler, W. Hefner, K. Buckl, H. Meinass, A. Meiswinkel, H.-J. Wernicke, G. Ebersberg, R. Müller, J. Bässler, H. Behringer, D. Mayer, Ullmann's Encyclopedia of Industrial Chemistry, Wiley-VCH: Weinheim, Germany, 2000.

[8] M. Jiang, X. Cui, L. Yang, Q. Yang, Z. Zhang, Y. Yang, H. Xing, Chem. Eng. J. 2018, 352, 803–810.

[9] G. Duan, W. Q. Jin, R. Krishna, Inorg. Chem. 2015, 54, 4279–4284. 25884592 10.1021/ic5030058

[10] X. Duan, Q. Zhang, J. F. Cai, Y. Yang, Y. J. Cui, Y. B. He, C. D. Wu, R. Krishna, B. L. Chen, G. D. Qian, J. Mater. Chem. A 2014, 2, 2628–2633.

[11] B. Lin, L. Li, H. Wu, H. Arman, B. Li, R.-G. Lin, W. Zhou, B. Chen, J. Am. Chem. Soc. 2017, 139, 8022–8028. 28574717 10.1021/jacs.7b03850

[12] M. L. Foo, R. Matsuda, Y. Hijikata, R. Krishna, H. Sato, S. Horike, A. Hori, J. Dua, Y. Sato, Y. Kubota, M. Takata, S. Kitagawa, J. Am. Chem. Soc. 2016, 138, 9, 3022–3030. 10.1021/jacs.5b10491 26876504

[13] W. Fan, S. Yuan, W. Wang, L. Feng, X. Liu, X. Zhang, X. Wang, Z. Kang, F. Dia, D. Yuan, D. Sun, H. C. Zhou, J. Am. Chem. Soc. 2020, 142, 8728–8737. 32188245 10.1021/jacs.0c00805

[14] Y. Hu, S. Xiang, W. Zhang, Z. Zhang, L. Wang, J. Bai, B. Chen, Chem. Commun. 2009, 45, 7551–7553. 10.1039/b917046d 20024276

[15] D. Humby, O. Benson, G. L. Smith, S. P. Argent, I. da Silva, Y. Cheng, S. Rudic, P. Manuel, M. D. Frogley, G. Cinque, L. K. Saunders, I. J. Vitorica-Yrezabal, G. F. S. Whitehead, T. L. Easun, W. Lewis, A. J. Blake, A. J. Ramirez-Cuesta, S. Yang, M. Schroder, Chem. Sci. 2019, 10, 1098–1106. 30774907 10.1039/c8sc03622e PMC6346404

[16] V. Colombo, S. Galli, H. J. Choi, G. D. Han, A. Maspero, G. Palmisano, N. Masciocchi, J. R. Long, Chem. Sci. 2011, 2, 1311–1319.

[17] N. M. Padial, E. Q. Procopio, C. Montoro, E. Lopez, J. E. Oltra, V. Colombo, A. Maspero, N. Masciocchi, S. Galli, I. Senkovska, S. Kaskel, E. Barea, J. A. R. Navarro, Angew. Chem. 2013, 125, 8448–8452; 10.1002/anie.201303484 23804226

[18] S. Dissegna, K. Epp, K. W. R. Heinz, G. Kieslich, R. A. Fischer, Adv. Mater. 2018, 30, 1704501. 10.1002/adma.201704501 29363822

[19] E. Lopez-Maya, C. Montoro, V. Colombo, E. Barea, J. A. R. Navarro, Adv. Funct. Mater. 2014, 24, 6130–6135.

[20] M. L. Rodríguez-Albelo, E. López-Maya, S. Hamad, A. R. Ruiz-Salvador, S. Calero, J. A. R. Navarro, Nat. Commun. 2017, 8, 14457. 28198376 10.1038/ncomms14457 PMC5316851

[21] M. I. Hossain, J. D. Cunningham, T. M. Becker, B. E. Grabicka, K. S. Walton, B. D. Rabideau, T. G. Glover, Chem. Eng. Sci. 2019, 203, 346–357.

[22] E. Q. Procopio, F. Linares, C. Montoro, V. Colombo, A. Maspero, E. Barea, J. A. R. Navarro, Angew. Chem. 2010, 122, 7466–7469; 10.1002/anie.201003314 20734368

[23] A. Hazra, A. Jain, M. S. Deenadayalan, S. A. Adalikwu, T. K. Maji, Inorg. Chem. 2020, 59, 9055–9064. 32515587 10.1021/acs.inorgchem.0c00932

[24] J. Gao, X. Qian, R−B. Lin, R. Krishna, H. Wu, W. Zhou, B. Chen, Angew. Chem. 2020, 132, 4426–4430; 10.1002/anie.202000323 PMC7935436 31944515

[25] S. M. F. Vilela, J. A. R. Navarro, P. Barbosa, R. F. Mendes, G. Pérez-Sánchez, H. Nowell, D. Ananias, F. Figueiredo, J. R. B. Gomes, J. P. C. Tomé, F. A. Almeida Paz, J. Am. Chem. Soc. 2021, 143, 3, 1365–1376. 10.1021/jacs.0c10421 33433193

[26] Z. Niu, X. Cui, T. Pham, G. Verma, P. C. Lan, C. Shan, H. Xing, K. A. Forrest, S. Suepaul, B. Space, A. Nafady, A. M. Al-Enizi, S. Ma, Angew. Chem. Int. Ed. 2021, 133, 5343–5348; 10.1002/anie.202016225 33403811

[27] W. Yoon, J. S. Lee, S. Lee, K. H. Cho, Y. K. Hwang, M. Daturi, C. H. Jun, R. Krishna, J. S. Chang, Chem. Eur. J. 2015, 21, 18431–18438. 26515022 10.1002/chem.201502893

[28] F. Luo, C. S. Yan, L. L. Dang, R. Krishna, W. Zhou, H. Wu, X. L. Dong, Y. Han, T. L. Hu, M. O'Keeffe, L. L. Wang, M. B. Luo, R. B. Lin, B. L. Chen, J. Am. Chem. Soc. 2016, 138, 5678–5684. 27113684 10.1021/jacs.6b02030

[29] R.-B. Lin, L. Li, H. Wu, H. Arman, B. Li, R.-G. Lin, W. Zhou, B. Chen, J. Am. Chem. Soc. 2017, 139, 8022–8028. 28574717 10.1021/jacs.7b03850

[30] Z. Yao, Z. Zhang, L. Liu, Z. Li, W. Zhou, Y. Zhao, Y. Han, B. Chen, R. Krishna, S. Xiang, Chem. Eur. J. 2016, 22, 5676–5683. 26934040 10.1002/chem.201505107

[31] K. J. Chen, H. S. Scott, D. G. Madden, T. Pham, A. Kumar, A. Bajpai, M. Lusi, K. A. Forrest, B. Space, J. J. Perry, M. J. Zaworotko, Chem 2016, 1, 753–765.

[32] J. Lee, C. Y. Chuah, J. Kim, Y. Kim, N. Ko, Y. Seo, K. Kim, T. H. Bae, E. Lee, Angew. Chem. Int. Ed. 2018, 57, 7869–7873. 10.1002/anie.201804442 29691972

[33] T. Qazvini, R. Babarao, S. G. Telfer, Chem. Mater. 2019, 31, 4919–4926.

[34] H.-M. Wen, C. Liao, L. Li, L. Yang, J. Wang, L. Huang, B. Li, B. Chen, J. Hu, Chem. Commun. 2019, 55, 11354–11357. 10.1039/c9cc05997k 31483423

[35] L. Peng, T. Pham, P. Li, T. Wang, Y. Chen, K. J. Chen, K. A. Forrest, B. Space, P. Cheng, M. J. Zaworotko, Z. Zhang, Angew. Chem. Int. Ed. 2018, 132, 11137–11141;

[36] R. Matsuda, R. Kitaura, S. Kitagawa, Y. Kubota, R. V. Belosludov, T. C. Kobayashi, H. Sakamoto, T. Chiba, M. Takata, Y. Kawazoe, Y. Mita, Nature 2005, 436, 238–241. 16015325 10.1038/nature03852

[37] L. Myers, J. M. Prausnitz, AIChE J. 1965, 11, 121–127.

[38] V. Colombo, C. Montoro, A. Maspero, G. Palmisano, N. Masciocchi, S. Galli, E. Barea, J. A. R. Navarro, J. Am. Chem. Soc. 2012, 134, 12830–12843. 22765315 10.1021/ja305267m

[39] https://www.3ds.com/products-services/biovia/products/molecular-modeling-simulation/biovia-materials-studio/.

