# Supplementary material for: Impact of Pore Size and Defects on the Selective Adsorption of Acetylene in Alkyne‐Functionalized Nickel(II)‐Pyrazolate‐Based MOFs
Source: Chemistry. 2021 Jul 9;27(46):11837–44. doi: 10.1002/chem.202100821 (PMC8457162; doi:10.1002/chem.202100821)
Supplement: Supplementary file 1 — Supporting Information [file CHEM-27-11837-s001.pdf]

Chemistry–A European Journal

Supporting Information

Impact of Pore Size and Defects on the Selective Adsorption of Acetylene in Alkyne-Functionalized Nickel(II)-Pyrazolate-Based MOFs

Farzaneh Afshariazar, Ali Morsali, Simona Sorbara, Natalia M. Padial, Esther Roldan-Molina, J. Enrique Oltra, Valentina Colombo, and Jorge A. R. Navarro*

Table of contents

S1. XRPD characterization S3

S2. Static adsorption measurements S5

S3. IAST theory studies S9

S4. Dynamic breakthrough adsorption studies S14

S5. Variable temperature pulse gas chromatography studies S17

S6. Synthesis of the organic linkers S23

S7. Elemental analysis S25

S8. Thermogravimetric analysis S26

S7. References S27

S8. Cif files of modellized Ni-MOFs systems loaded with C_2H_2 and CO_2 guest molecules (Separate ESI files).

S1. XRPD characterization

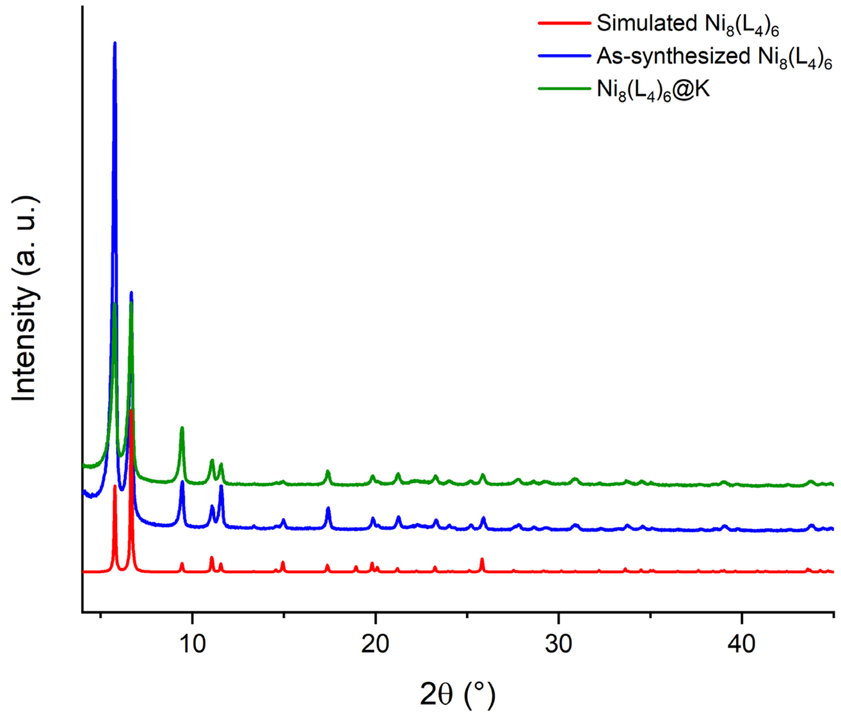

Figure S1. PXRD patterns of [$Ni_8(L_4)_6$] and [$Ni_8(L_4)_6$]@K.

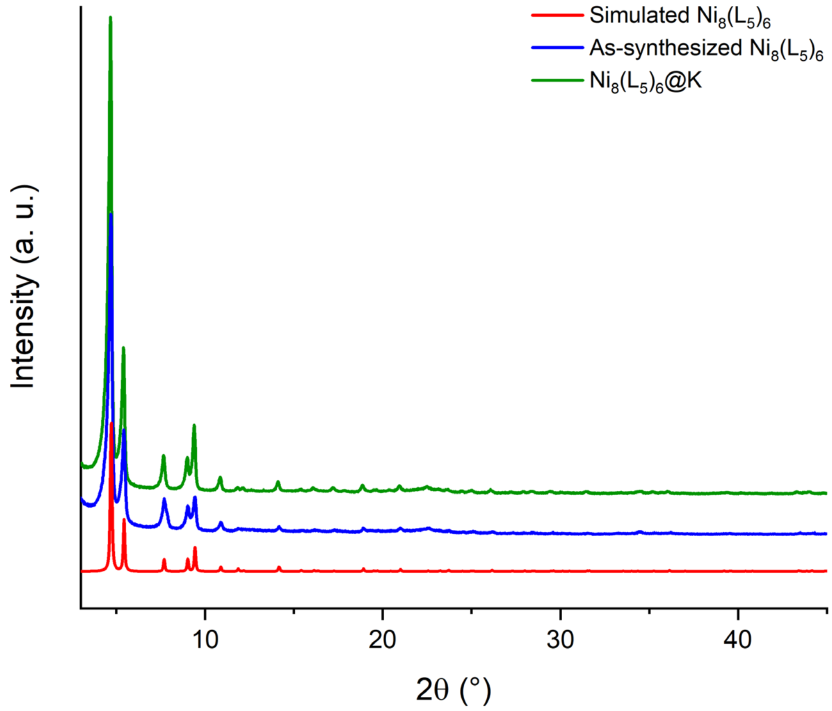

Figure S2. PXRD patterns for [$Ni_8(L_5)_6$] and [$Ni_8(L_5)_6$]@K.

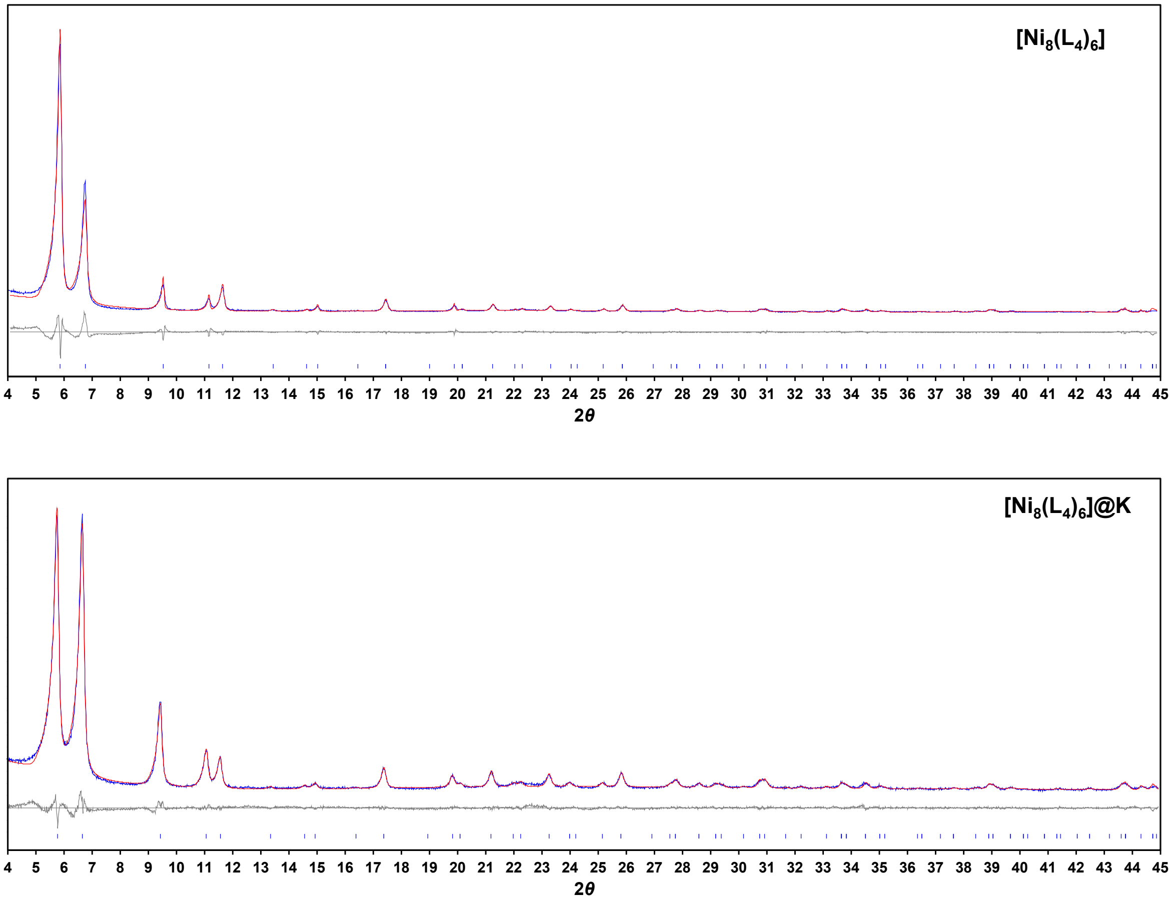

Figure S3. Structureless Le Bail refinements as obtained for $[Ni_8(L_4)_6]$ (Cubic, *Fm-3m*, a = 26.4237(7) Å) and $[Ni_8(L_4)_6]$@K (Cubic, *F*m-3m, a = 26.4497(6) Å). Blue line: experimental data; red line: calculated profile; gray line: difference between experimental and calculated profiles; blue tick marks: peaks positions. Rwp 10.47 and 7.66, respectively.

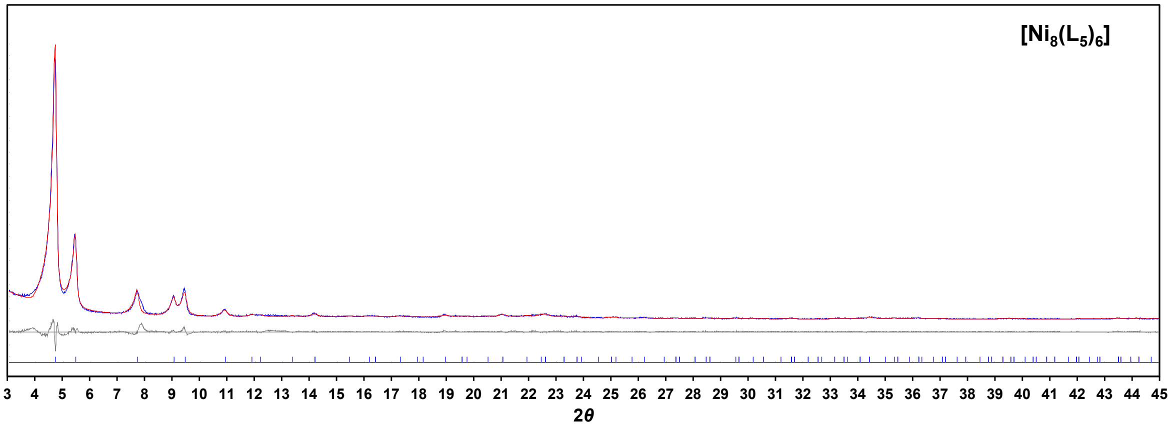

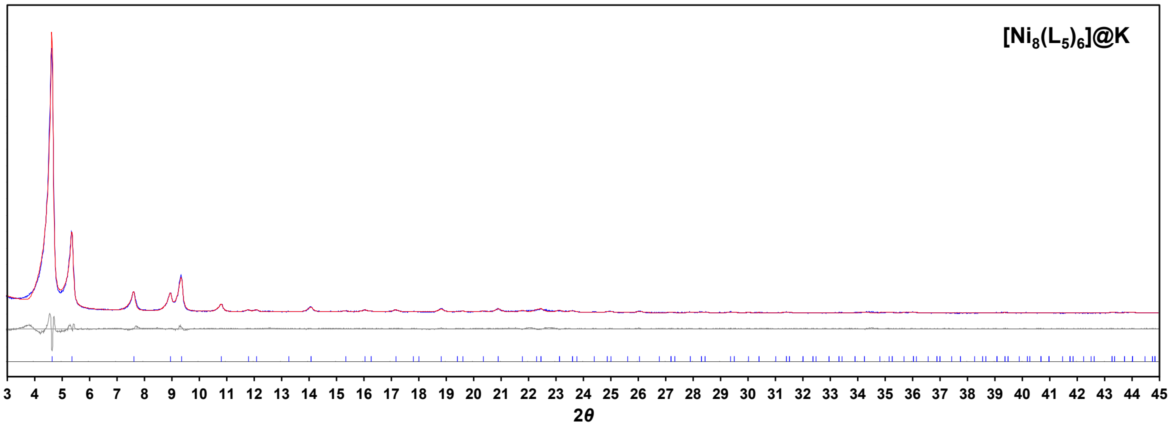

Figure S4. Structureless Le Bail refinements as obtained for [$Ni_8(L_5)_6$] (Cubic, *Fm-3m*, a = 32.3915(2) Å) and [$Ni_8(L_5)_6$]@K (Cubic, *F*m-3m, a = 32.5508(2) Å). Blue line: experimental data; red line: calculated profile; gray line: difference between experimental and calculated profiles; blue tick marks: peaks positions. Rwp 8.22 and 7.66, respectively.

S2. Static adsorption measurements

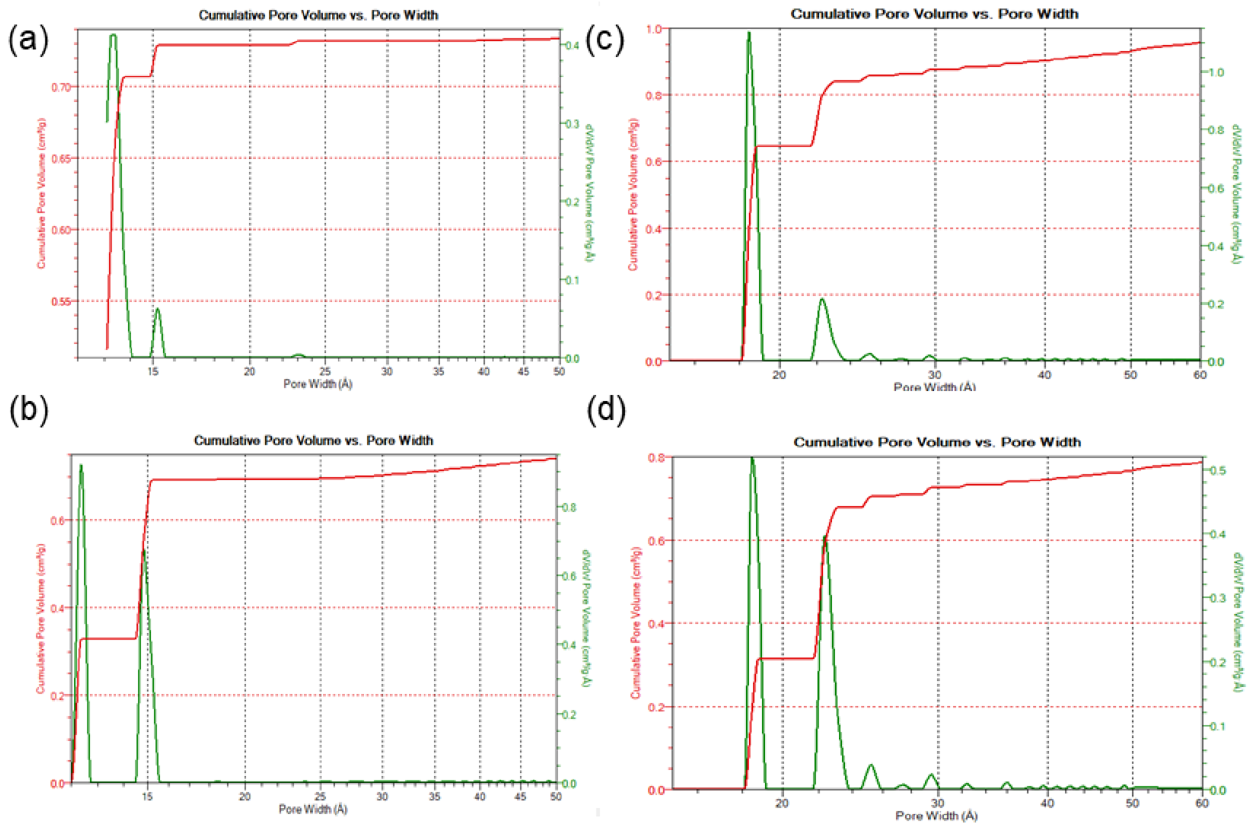

Figure S5. Impact of defect creation on the $[Ni_8(L)_6]$ systems on pore accessibility. Calculated DFT pore size distribution from N_2 adsorption isotherms at 77 K for $[Ni_8(L_4)_6]$ (a), $[Ni_8(L_4)_6]$@K (b), $[Ni_8(L_5)_6]$ (c), $[Ni_8(L_5)_6]$@K (d).

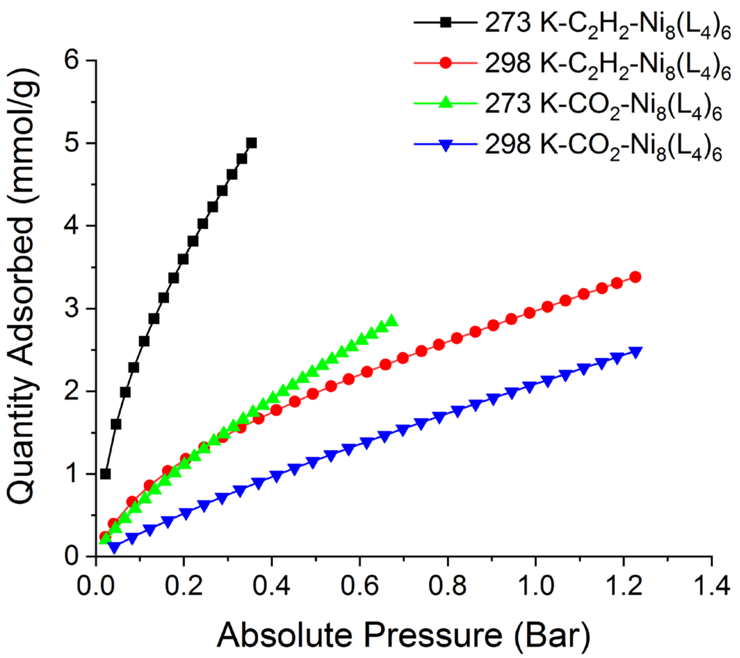

Figure S6. Gas sorption isotherms of $Ni_8(L_4)_6$ for C_2H_2 and CO_2 at 273 K and 298 K.

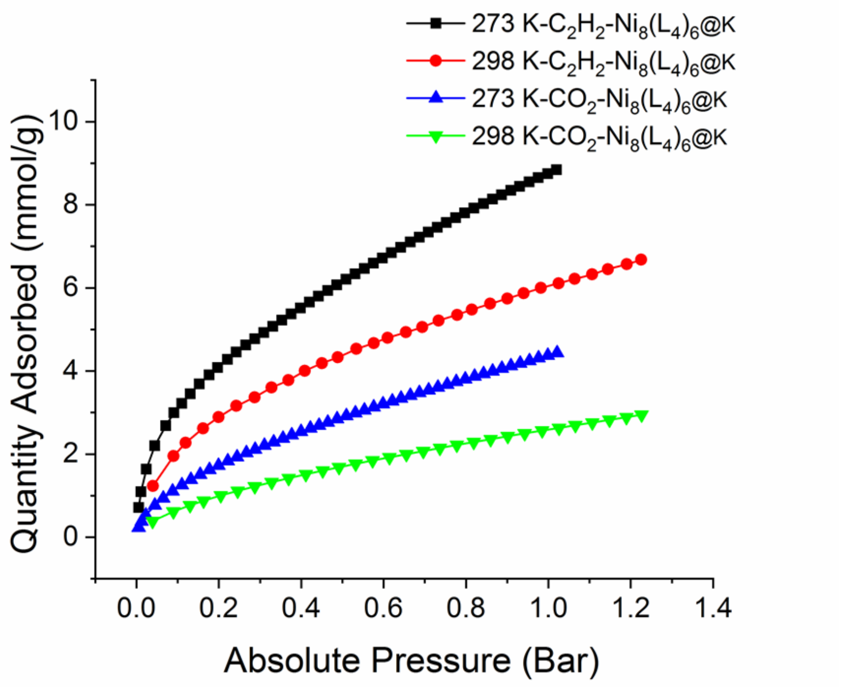

Figure S7. Gas sorption isotherm of $Ni_8(L_4)_6$@K for C_2H_2 and CO_2 at 273 K and 298 K.

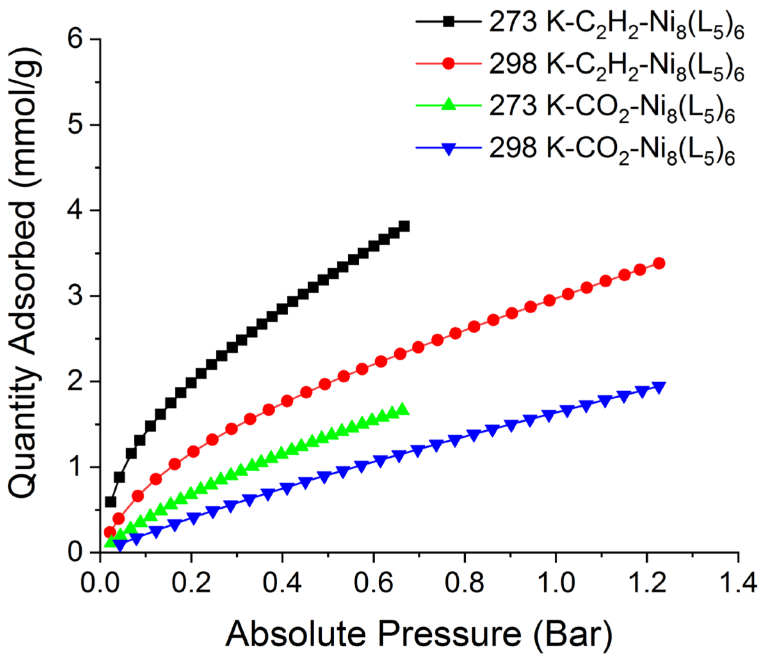

Figure S8. Gas sorption isotherms of $Ni_8(L_5)_6$ for C_2H_2 and CO_2 at 273 K and 298 K.

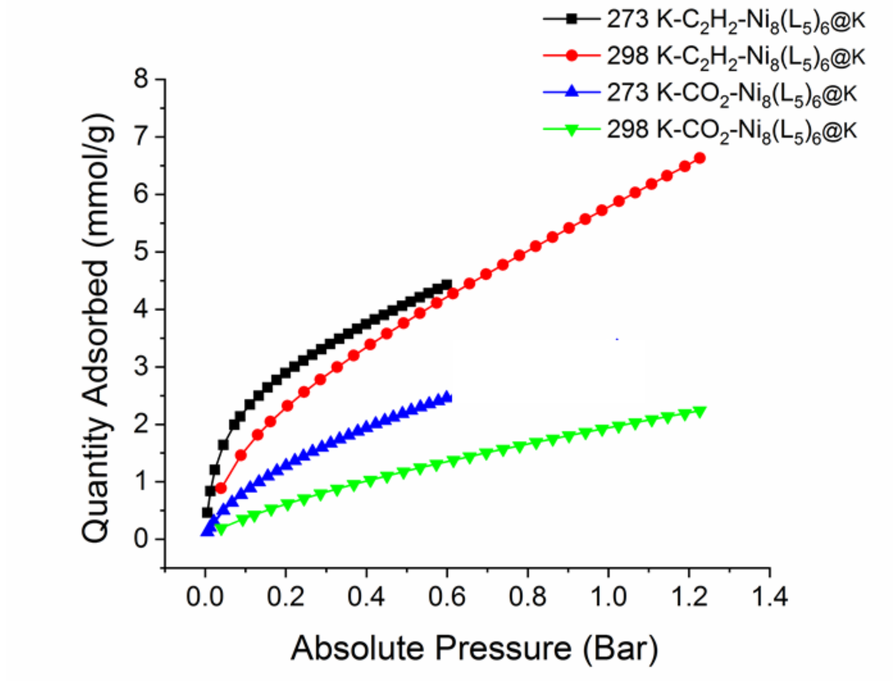

Figure S9. Gas sorption isotherms of $Ni_8(L_5)_6$@K for C_2H_2 and CO_2 at 273 K and 298 K.

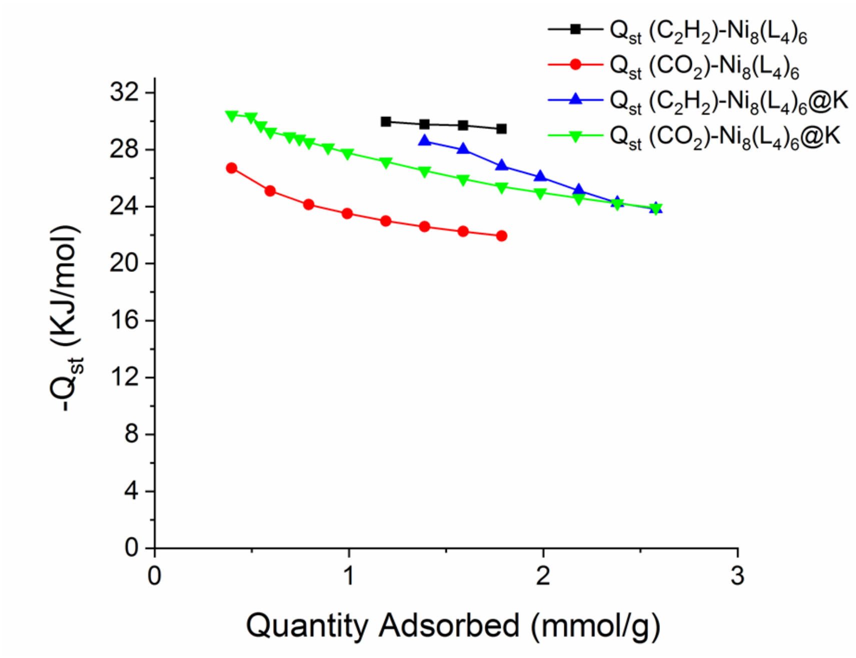

Figure S10. Isosteric heats of adsorption as a function of the gas uptake for $Ni_8(L_4)_6$ and $Ni_8(L_4)_6$@K, respectively.

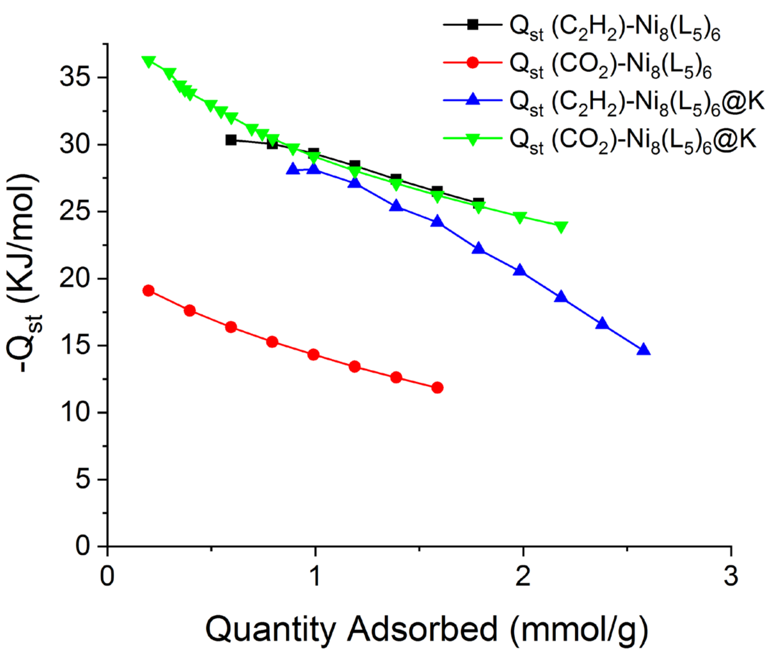

Figure S11. Isosteric heats of adsorption as a function of the gas uptake for $Ni_8(L_5)_6$ and $Ni_8(L_5)_6$@K, respectively.

S3. IAST theory studies

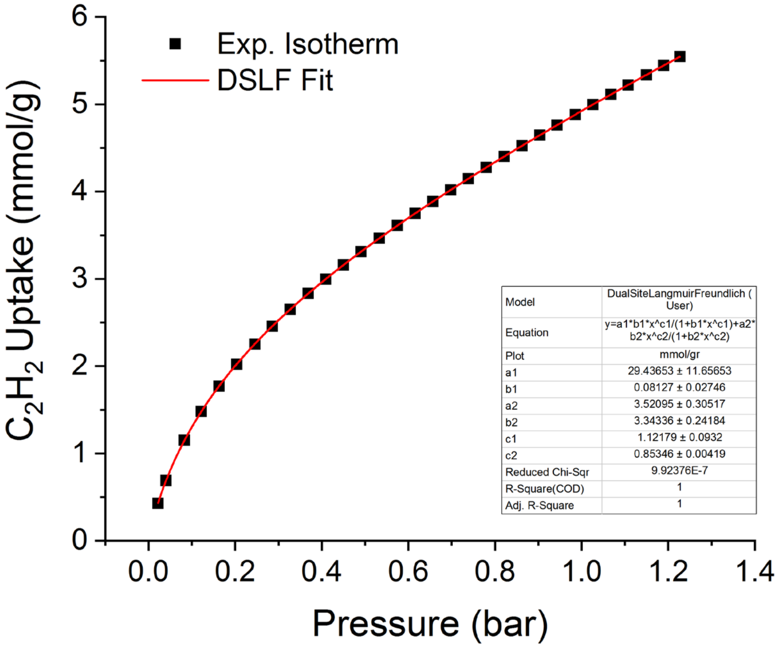

Figure S12. The dual-site Langmuir-Freundlich fitting of 298 K C_2H_2 sorption data for $Ni_8(L_4)_6$.

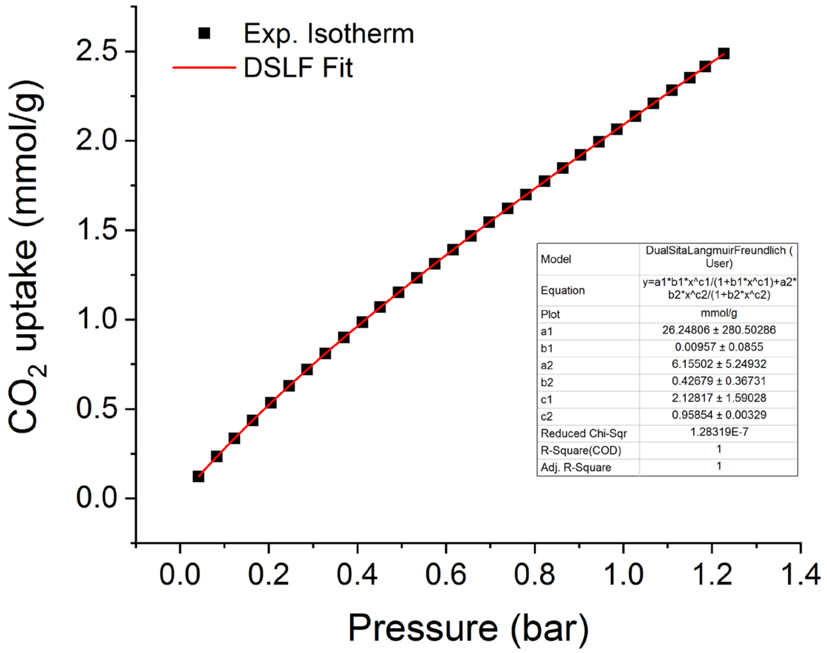

Figure S13. The dual-site Langmuir-Freundlich fitting of 298 K CO_2 sorption data for $Ni_8(L_4)_6$.

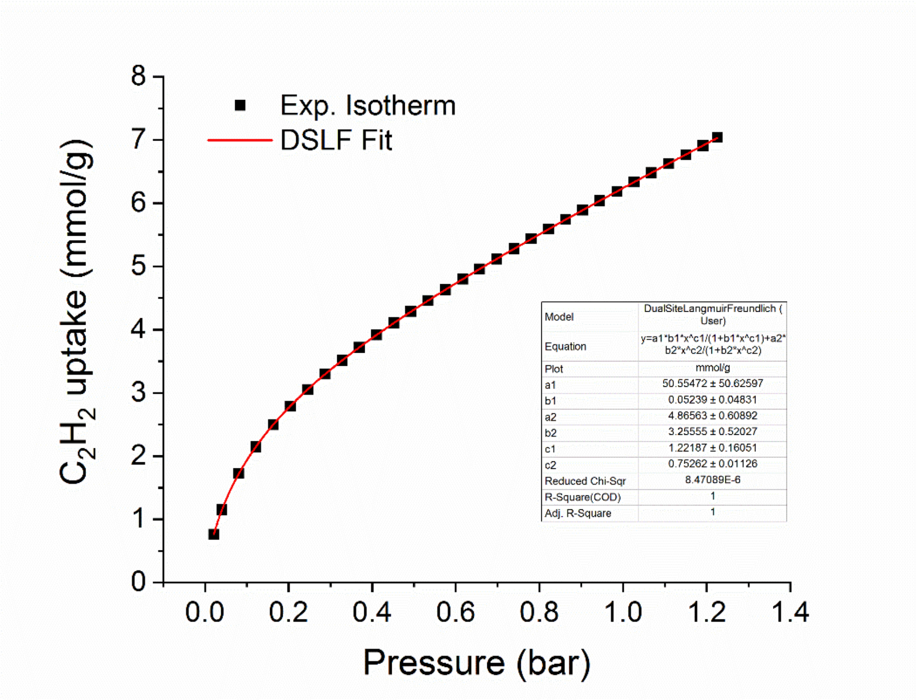

Figure S14. The dual-site Langmuir-Freundlich fitting of 298 K C_2H_2 sorption data for $Ni_8(L_4)_6$@K.

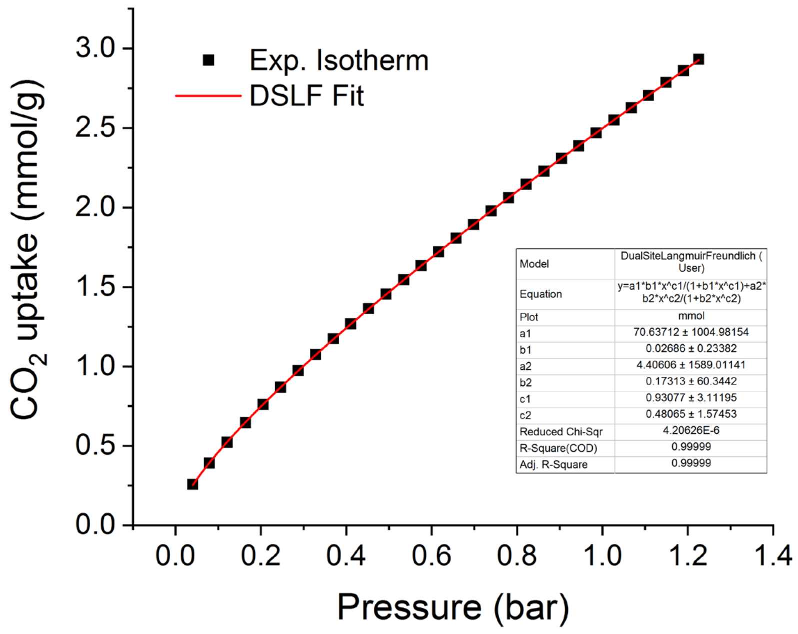

Figure S15. The dual-site Langmuir-Freundlich fitting of 298 K CO_2 sorption data for $Ni_8(L_4)_6$@K.

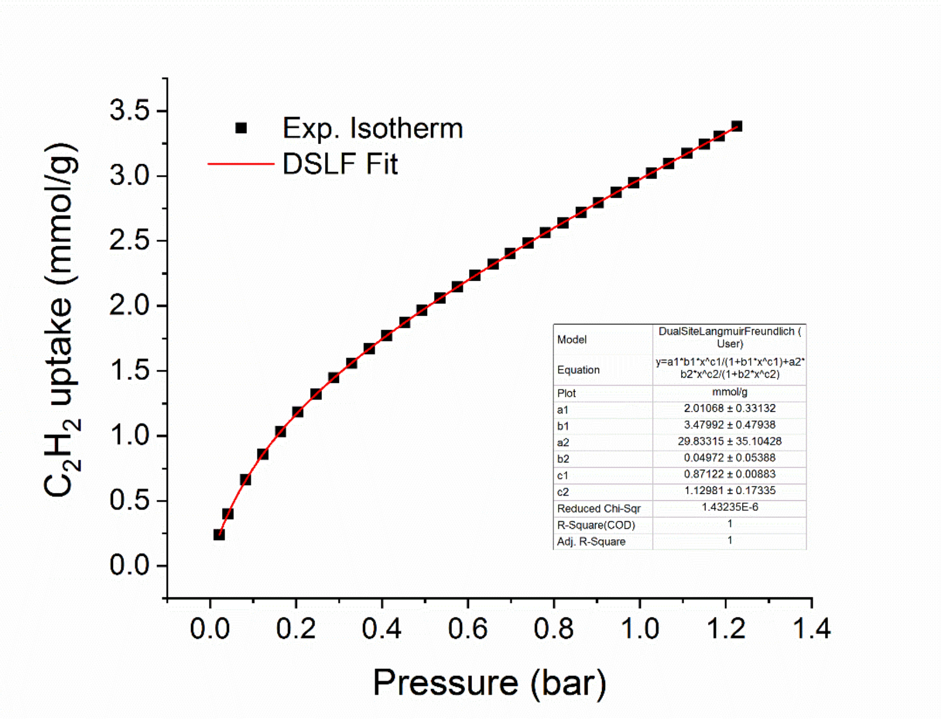

Figure S16. The dual-site Langmuir-Freundlich fitting of 298 K C_2H_2 sorption data for $Ni_8(L_5)_6$.

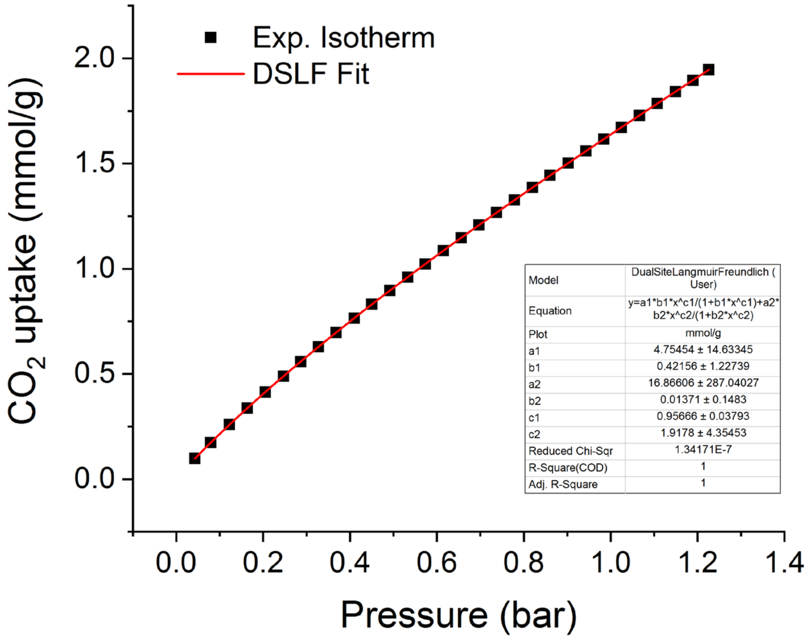

Figure S17. The dual-site Langmuir-Freundlich fitting of 298 K CO_2 sorption data for $Ni_8(L_5)_6$.

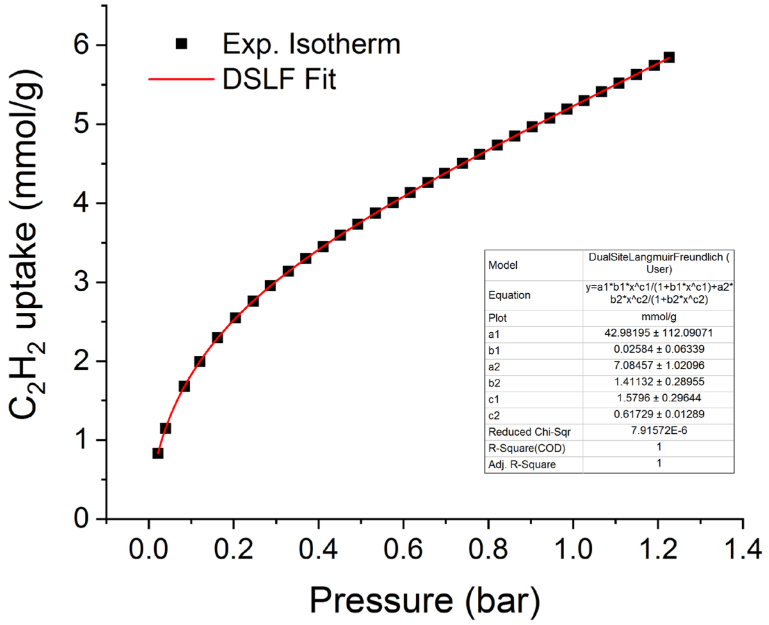

Figure S18. The dual-site Langmuir-Freundlich fitting of 298 K C_2H_2 sorption data for $Ni_8(L_5)_6$@K.

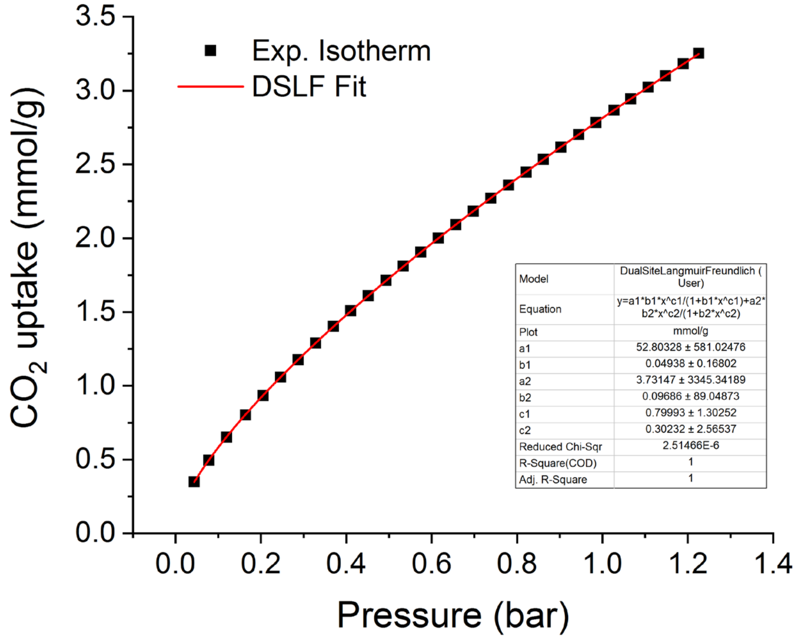

Figure S19. The dual-site Langmuir-Freundlich fitting of 298 K CO_2 sorption data for $Ni_8(L_5)_6$@K.

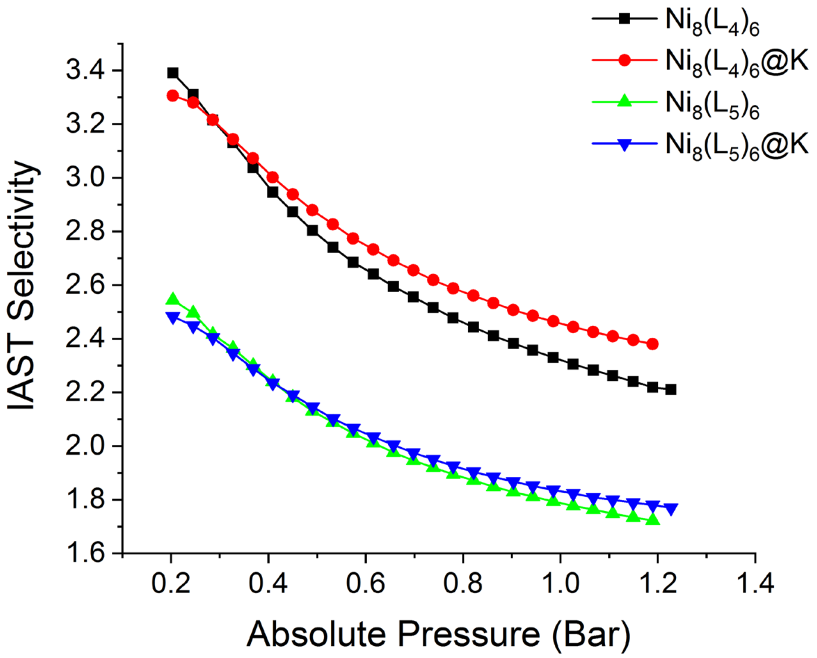

Figure S20. Predicted adsorption selectivity for C_2H_2 over CO_2 for Ni-MOF structures calculated by employing the IAST theory at 298 K.

S4. Dynamic breakthrough adsorption studies

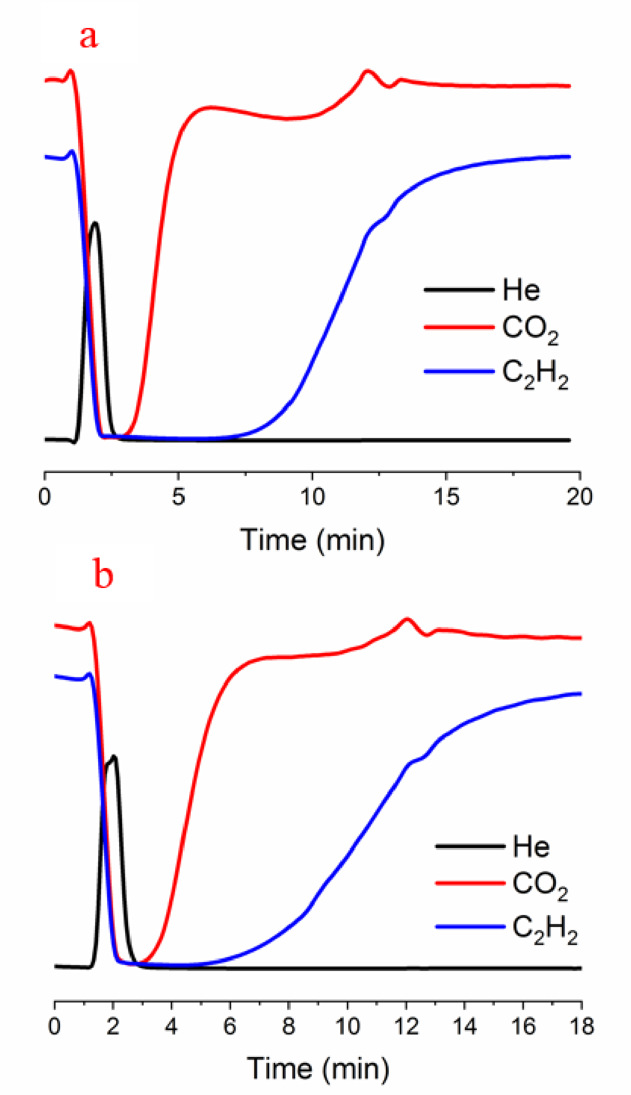

Figure S21. Breakthrough curves of $[Ni_8(L_4)_6]$: a) 273 K, b) 298 K for a gas mixture of $C_2H_2/CO_2/N_2$ (3:3:14 $mLmin^{-1}$).

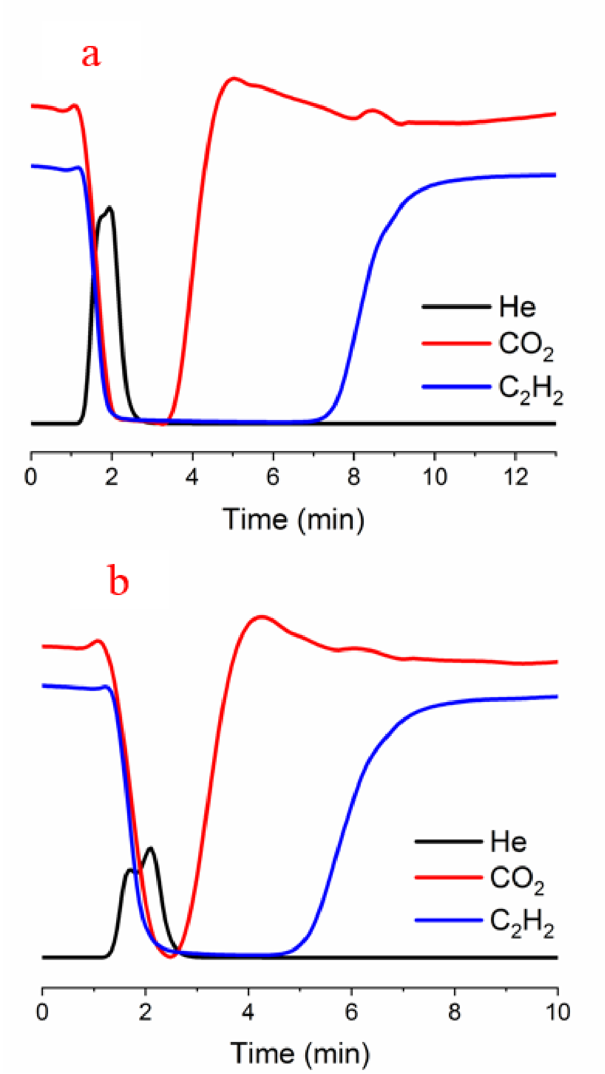

Figure S22. Breakthrough curves of $[Ni_8(L_5)_6]$: a) 273 K, b) 298 K for a gas mixture of $C_2H_2/CO_2/N_2$ (3:3:14 $mLmin^{-1}$).

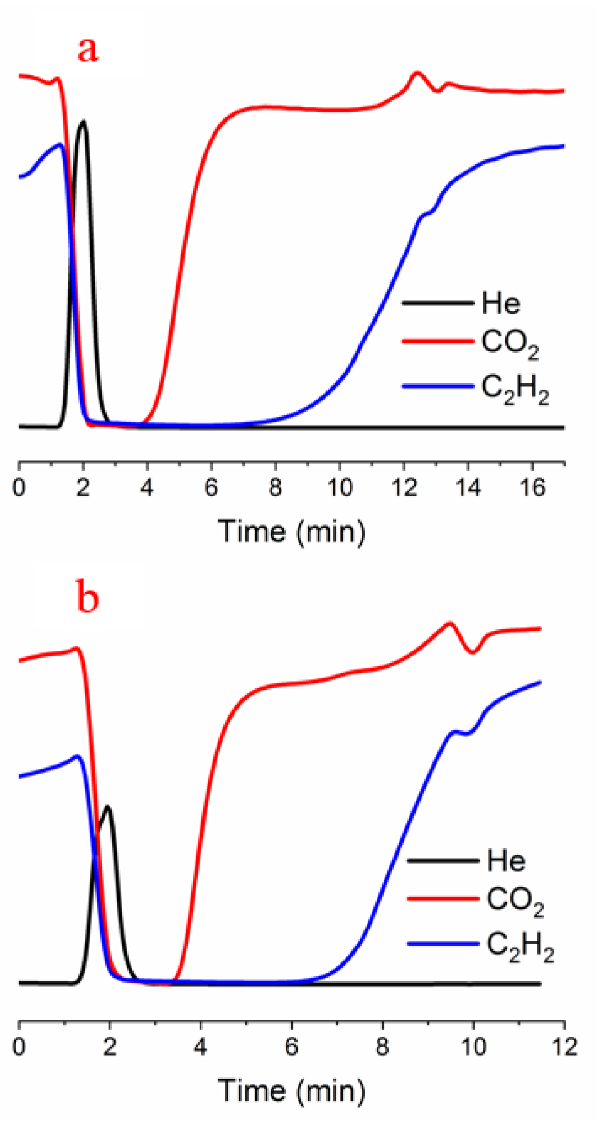

Figure S23. Breakthrough curves of $[Ni_8(L_5)_6]$@K: a) 273 K, b) 298 K for a gas mixture of $C_2H_2/CO_2/N_2$ (3:3:14 $mLmin^{-1}$).

S5. Variable temperature pulse gas chromatography studies

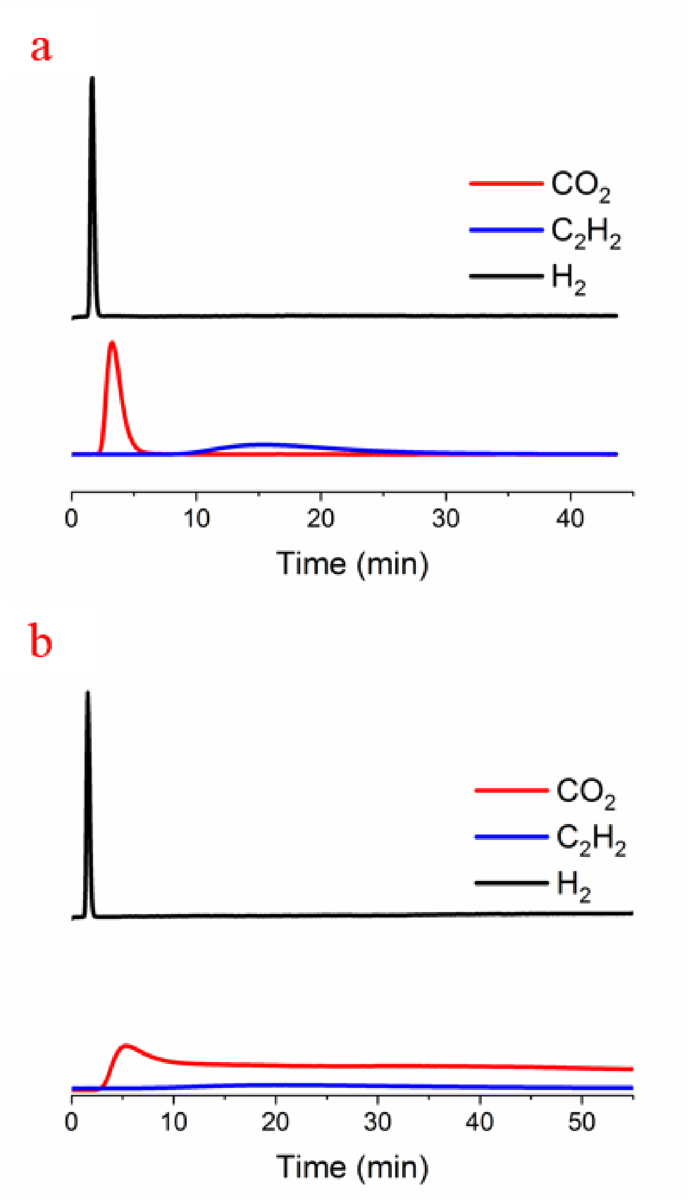

Figure S24. Pulse gas chromatographic curves for C_2H_2, CO_2, and H_2 gas molecules, a) $[Ni_8(L_4)_6]$, b) $[Ni_8(L_4)_6]$@K at 298 K.

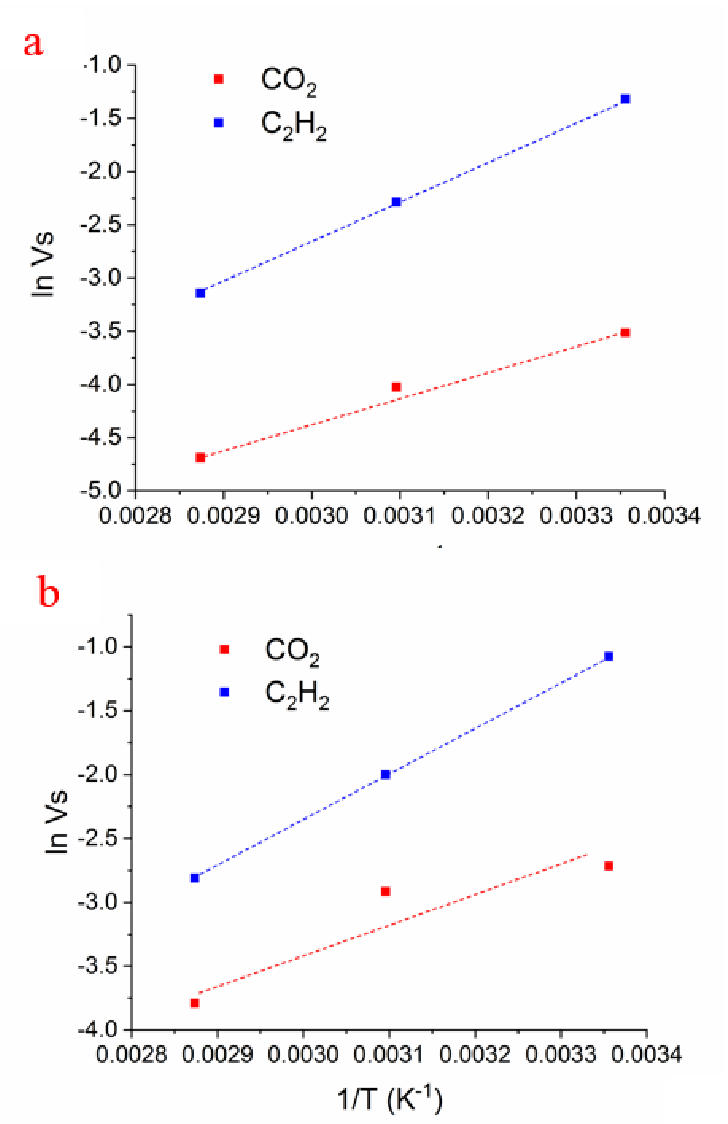

Figure S25. Fitting diagrams of the variation of the Vg (cm^3g^{-1}) as a function of the various temperatures (273–248 K) for a) $Ni_8(L_4)_6$, and b) $Ni_8(L_4)_6$@K.

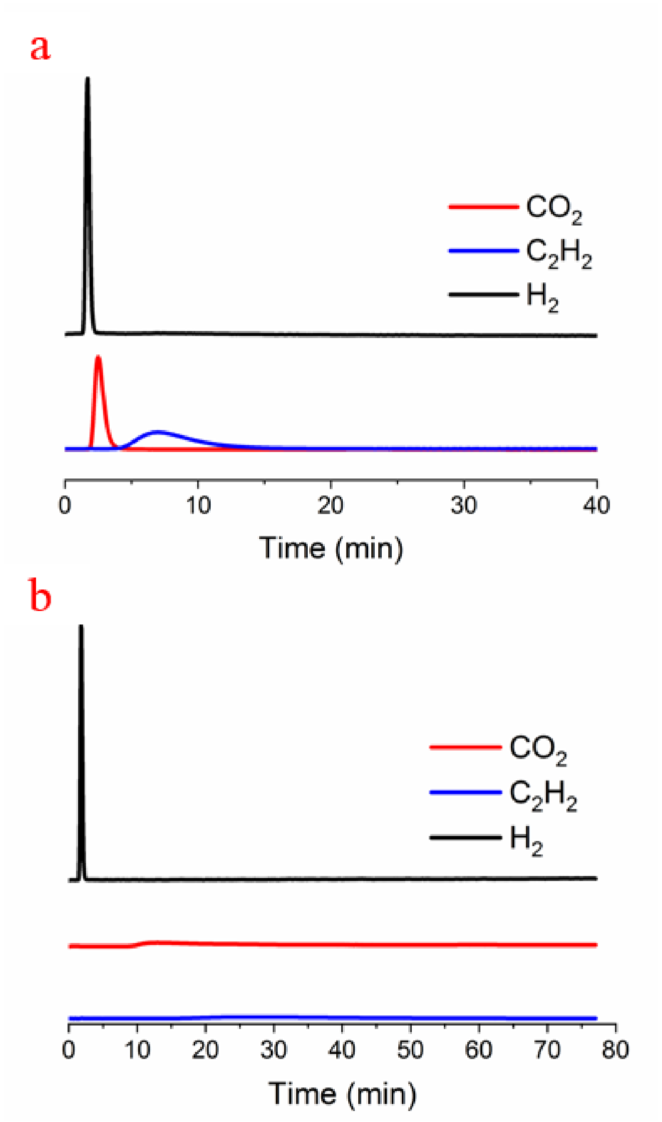

Figure S26. Pulse gas chromatographic curves for C_2H_2, CO_2, and H_2 gas molecules, a) [$Ni_8(L_5)_6$], b) [$Ni_8(L_5)_6$]@K at 298 K.

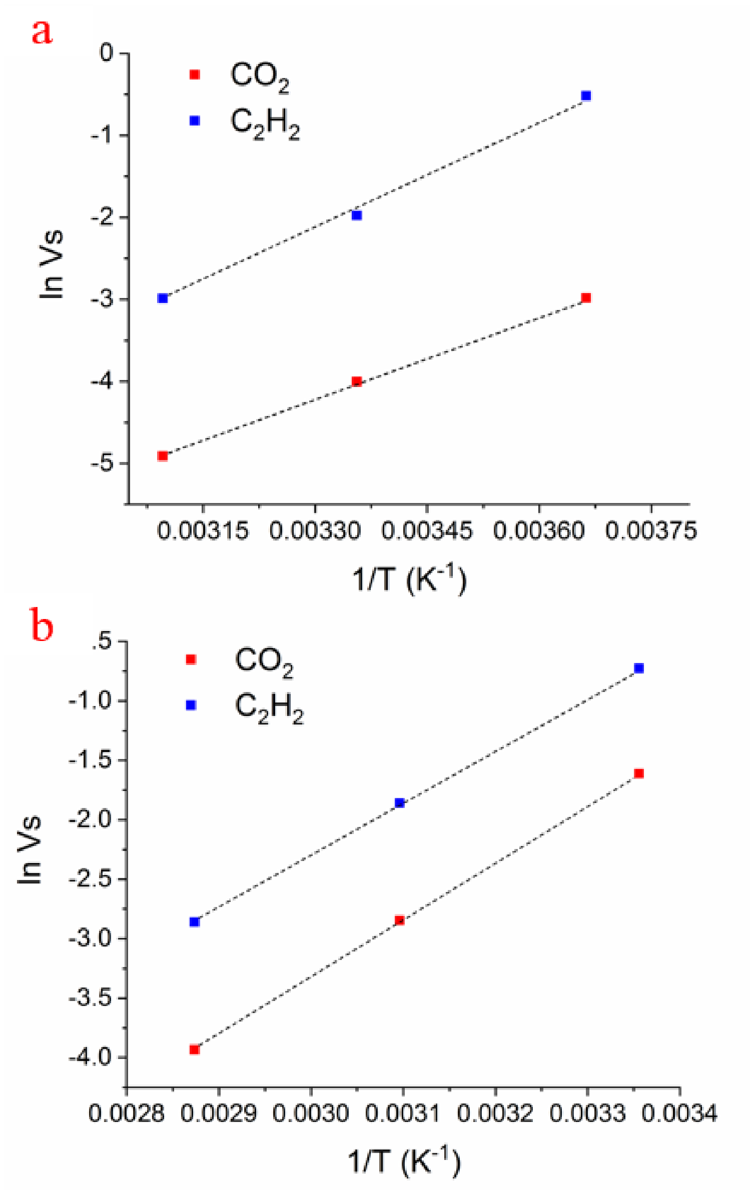

Figure S27. Fitting diagrams of the variation of the Vg (cm^3g^{-1}) as a function of the various temperatures (273–248 K) for a) $Ni_8(L_5)_6$, and b) $Ni_8(L_5)_6$@K.

Table S1. Summary of thermodynamic data for Ni-MOFs derived from pulse gas chromatographic experiments.

**Adsorbate**	**$-\Delta H_{ads}$(kj.mol^{-1})**	**K_H (cm^{3}m^{-2})^a**	**$\alpha_{CO2/C2H2}$**	**$\alpha_{C2H2/CO2}$**
		$[Ni_8(L4)_6]$		
CO_2	12.2	0.04	0.148	
C_2H_2	28.7	0.27		6.75
		$Ni_8(L4)_6$@K		
CO_2	14.9	0.15	0.23	
C_2H_2	28.2	0.66		4.4
		$[Ni_8(L5)_6]$		
CO_2	17.2	0.03	0.002	
C_2H_2	33.7	0.17		5.67
		$Ni_8(L5)_6$@K		
CO_2	35.2	0.2	0.41	
C_2H_2	34.7	0.49		2.45

S6. Synthesis and characterization of the organic linkers.

L_4 and L_5 bipyrazolate ligands were prepared as Boc_2L_4 and Boc_2L_5 according to the previously reported procedure.[1] Boc-group was removed during the synthesis of Ni-MOFs without any additional step.

Boc$_2$L$_4$

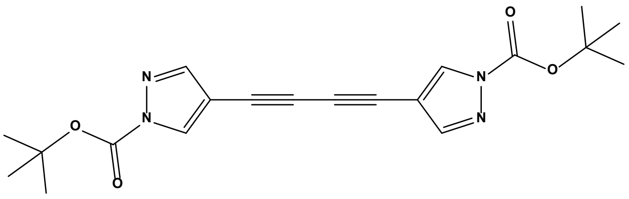

^{1}H NMR (400 MHz, $CDCl_3$) δ: 8.24 (s, 2H), 7.78 (s, 2H), 1.64 (s, 18H) ppm. ^{13}C NMR (100 MHz, $CDCl_3$) δ: 146.6 (C), 146.0 (CH), 134.4 (CH), 105.1(C), 86.6 (C), 76.1 (C), 71.6 (C), 27.9 (CH_3) ppm. Spectral data are in agreement with previously reported values.[1]

Boc$_2$L$_5$

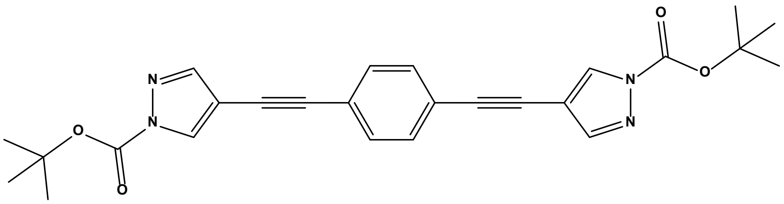

^{1}H NMR (400 MHz, $CDCl_3$) δ: 8.25 (s, 2H), 7.82 (s, 2H), 7.46 (s, 4H), 1.67 (s, 18H) ppm.^{13}C NMR (100 MHz, $CDCl_3$) δ: 146.9 (C), 145.6 (CH), 132.8 (CH), 131.4 (CH), 122.8 (C), 106.4 (C), 91.4 (C), 86.3 (C), 81.0 (C), 27.9 (CH_3) ppm. Spectral data are in agreement with previously reported values.[1]

^{1}H and ^{13}C NMR Spectra.

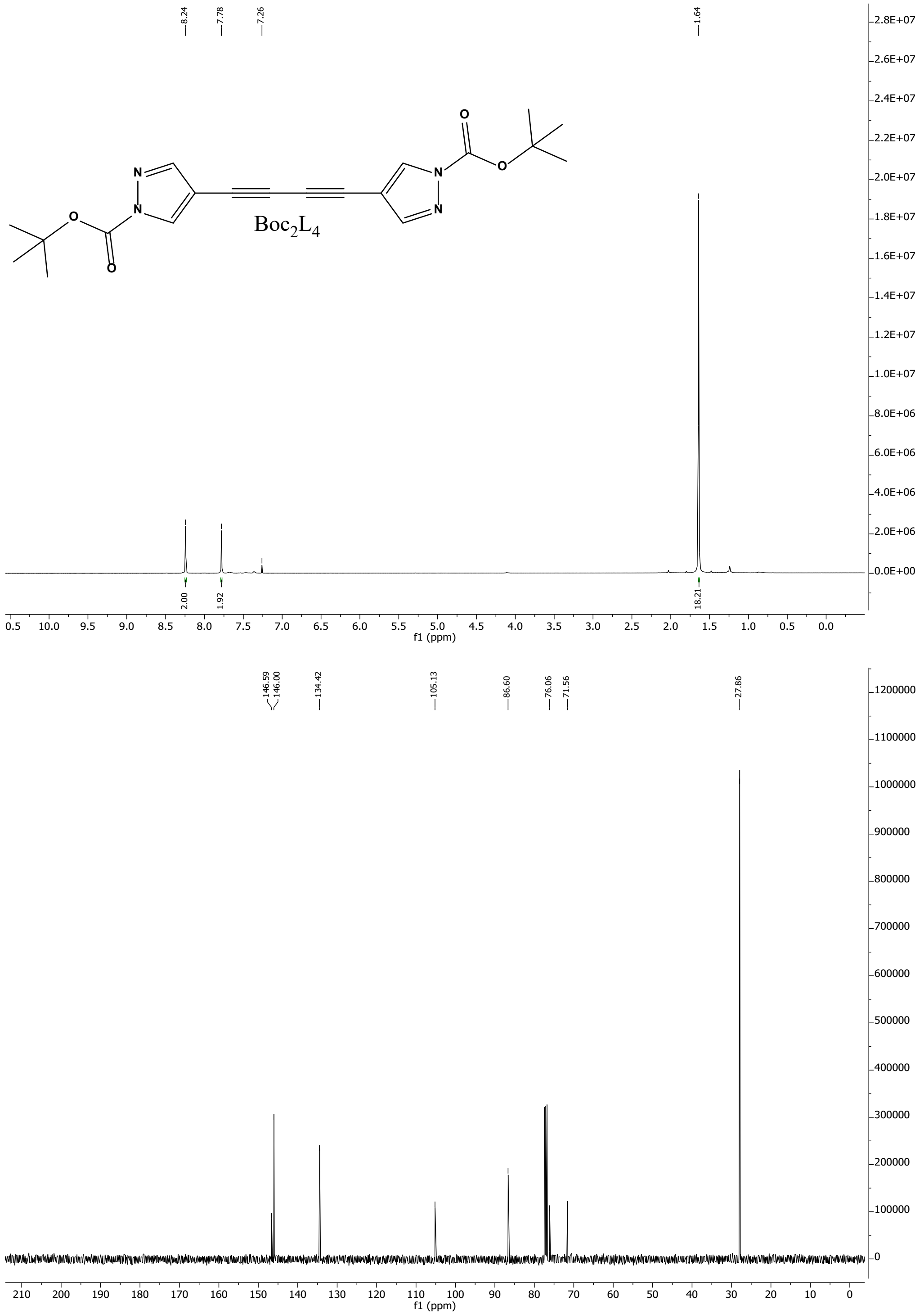

Figure S27. 1H NMR (top) and 13C NMR (bottom) for **Boc$_2$L$_4$**

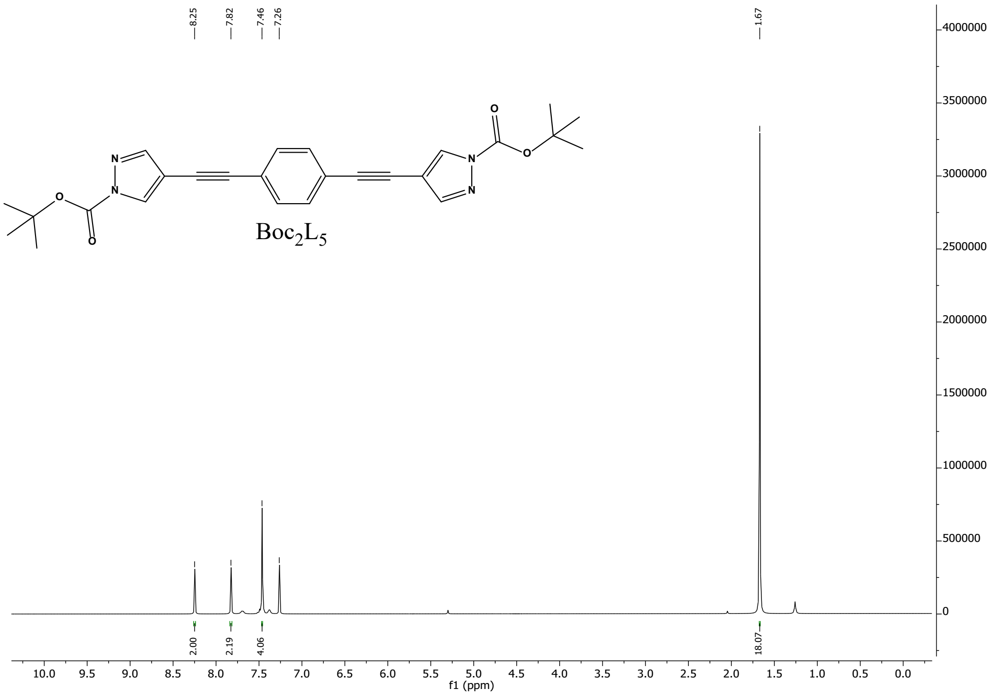

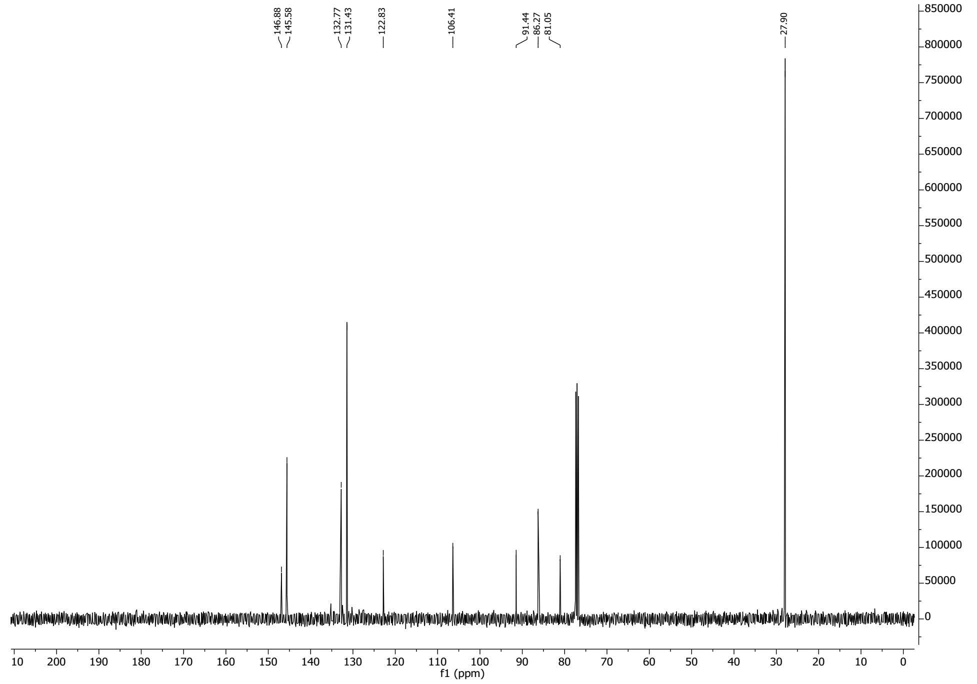

Figure S28. 1H NMR (top) and 13C NMR (bottom) for **Boc$_2$L$_5$**

S7. Elemental analysis

Table S2. EA of Ni-MOFs.

MOF Material	N	C	H
$[Ni_8(L_4)_6]$ (exp.)	12.721	30.60	3.80
$Ni_8(OH)_4(H_2O)_2(C_{10}N_4H_4)_6(H_2O)_{38}$ (calculated)	14.37	30.81	4.65
$[Ni_8(L_4)_6]$@K (exp.)	10.38	24.28	5.05
$K[Ni_8(OH)_4(H_2O)_2(C_{10}N_4H_4)_{5.5}](H_2O)_{60}$	11.49	24.63	5.56
$[Ni_8(L_5)_6]$ (exp.)	13.52	45.91	3.58
$Ni_8(OH)_4(H_2O)_2(C_{16}N_4H_8)_6(H_2O)_{22}$ (calculated)	13.46	45.98	4.02
$[Ni_8(L_5)_6]$@K (exp.)	12.17	43.28	3.23
$K[Ni_8(OH)_4(H_2O)_2(C_{16}N_4H_8)_{5.5}](H_2O)_{22}$ (calculated)	12.70	43.58	3.95

S8. Thermogravimetric analysis

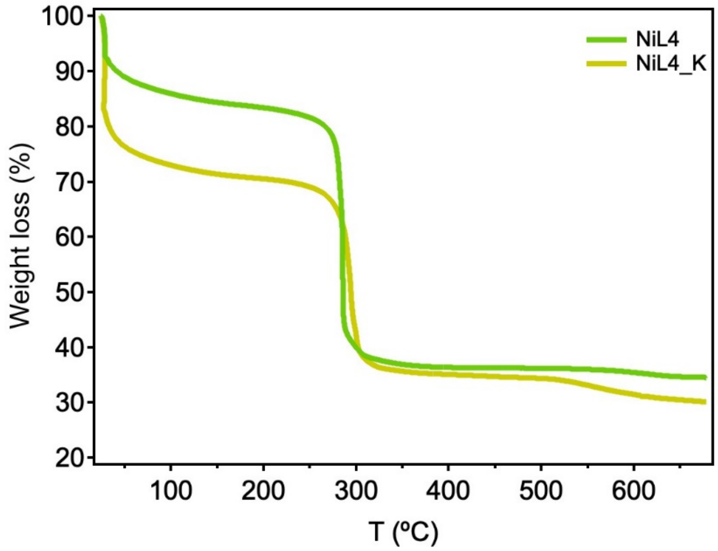

Figure S29. TGA for $[Ni_8(L_4)_6]$ and $[Ni_8(L_4)_6]$@K systems

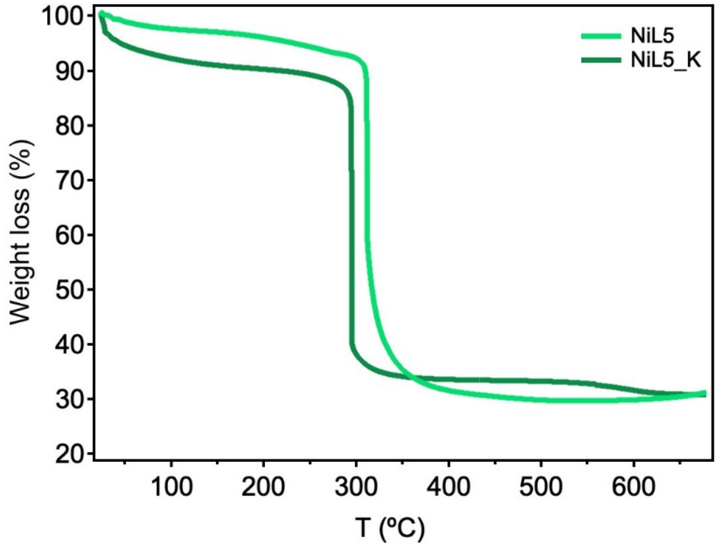

Figure S30. TGA for $[Ni_8(L_5)_6]$ and $[Ni_8(L_5)_6]$@K systems

S9. References

[1] N. M. Padial, E. Q. Procopio, C. Montoro, E. Lopez, J. E. Oltra, V. Colombo, A. Maspero, N. Masciocchi, S. Galli, I. Senkovska, S. Kaskel, E. Barea, J. A. R. Navarro, *Angew. Chem. Int. Ed.*, 2013, 52, 1-6. 8290-8294.
